# A Se Nanoparticle/MgFe‐LDH Composite Nanosheet as a Multifunctional Platform for Osteosarcoma Eradication, Antibacterial and Bone Reconstruction

**DOI:** 10.1002/advs.202403791

**Published:** 2024-07-03

**Authors:** Yixin Bian, Kexin Zhao, Tingting Hu, Chaoliang Tan, Ruizheng Liang, Xisheng Weng

**Affiliations:** ^1^ Department of Orthopedic Surgery State Key Laboratory of Complex Severe and Rare Diseases Peking Union Medical College Hospital Chinese Academy of Medical Science and Peking Union Medical College Beijing 100730 P. R. China; ^2^ State Key Laboratory of Chemical Resource Engineering Beijing Advanced Innovation Center for Soft Matter Science and Engineering Beijing University of Chemical Technology Beijing 100029 P. R. China; ^3^ Department Electrical and Electronic Engineering The University of Hong Kong Pokfulam Road Hong Kong SAR 999077 P. R. China; ^4^ Quzhou Institute for Innovation in Resource Chemical Engineering Quzhou 324000 P. R. China

**Keywords:** antibacterial, layered double hydroxides, osteogenesis, osteosarcoma, selenium nanoparticle

## Abstract

Despite advances in treating osteosarcoma, postoperative tumor recurrence, periprosthetic infection, and critical bone defects remain critical concerns. Herein, the growth of selenium nanoparticles (SeNPs) onto MgFe‐LDH nanosheets (LDH) is reported to develop a multifunctional nanocomposite (LDH/Se) and further modification of the nanocomposite on a bioactive glass scaffold (BGS) to obtain a versatile platform (BGS@LDH/Se) for comprehensive postoperative osteosarcoma management. The uniform dispersion of negatively charged SeNPs on the LDH surface restrains toxicity‐inducing aggregation and inactivation, thus enhancing superoxide dismutase (SOD) activation and superoxide anion radical (·O_2_^−^)‐H_2_O_2_ conversion. Meanwhile, Fe^3+^ within the LDH nanosheets can be reduced to Fe^2+^ by depleting glutathione (GSH) in the tumor microenvironments (TME), which can catalyze H_2_O_2_ into highly toxic reactive oxygen species. More importantly, incorporating SeNPs significantly promotes the anti‐bacterial and osteogenic properties of BGS@LDH/Se. Thus, the developed BGS@LDH/Se platform can simultaneously inhibit tumor recurrence and periprosthetic infection as well as promote bone regeneration, thus holding great potential for postoperative “one‐stop‐shop” management of patients who need osteosarcoma resection and scaffold implantation.

## Graphical abstract

Selenium nanoparticle‐decorated MgFe‐LDH nanosheets are loaded on a bioactive glass scaffold to develop a postoperative “one‐stop‐shop” therapeutic platform for osteosarcoma.

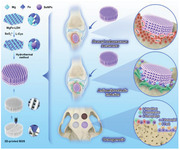

## 1 Introduction

Osteosarcoma is ranked as the second leading cause of tumor‐related mortality in adolescents.^[^ ^1^, ^2^, ^3^ ^]^ Currently, limb‐salvage tumor resection represents the main therapeutic strategy for osteosarcoma.^[^ ^4^, ^5^, ^6^, ^7^, ^8^, ^9^ ^]^ However, a dilemma arises in limb‐salvage tumor resection as expanding resection scope to mitigate postoperative recurrence entails extensive bone defects, while minimizing tumor resection extent raises concerns of osteosarcoma recurrence.^[^ ^10^, ^11^, ^12^ ^]^ Additionally, periprosthetic infections, especially those caused by drug‐resistant bacteria, can cause devastating prosthesis failure and induce refractory systemic inflammatory responses, underscoring significant menaces for frail osteosarcoma patients. Thus, a novel multifunctional implant that can simultaneously prevent tumor recurrence/periprosthetic infection and promote bone reconstruction is of great importance for improving the prognosis of osteosarcoma patients who need osteosarcoma resection and scaffold implantation. Recently, several scaffolds have been designed catering to this circumstance.^[^ ^13^, ^14^, ^15^, ^16^, ^17^, ^18^, ^19^, ^20^, ^21^, ^22^, ^23^ ^]^ For example, Huang et al.^[^ ^13^ ^]^ developed a gadolinium‐complex and molybdenum sulfide co‐doped N‐acryloyl glycinamide/gelatin methacrylate multifunctional hydrogel, of which the MoS_2_ endowed the scaffold with exceptional photothermal ability for tumor and bacterial killing, while the gadolinium‐complex simultaneously induced bone regeneration. However, bone tissue is typically covered by thick layers of muscle and skin, rendering shallow light penetration and significantly limiting the utility of photo‐based therapies. Conversely, chemodynamic therapy (CDT), activated by tumor microenvironment (TME) triggering Fenton or Fenton‐like reactions to translate H_2_O_2_ into hydroxyl radicals (·OH), shows great promise in precision tumor‐targeted therapy with no need for external stimuli.^[^ ^24^, ^25^, ^26^, ^27^ ^]^ For example, Pang et al.^[^ ^28^ ^]^ fabricated a Cu and Mn‐doped borosilicate nanoparticle, in which the bioactive Cu^2+^ and Mn^3+^ ions simultaneously promoted bone reconstruction and produced ·OH through Fenton‐like reaction to induce tumor cell apoptosis. Nevertheless, the efficiency of CDT is significantly compromised by limited H_2_O_2_ concentration in the TME. Moreover, most of the reported Fenton agents usually lack intrinsic antibacterial and osteogenic properties. Hence, developing a versatile scaffold coupling enhanced CDT efficiency with intrinsic antibacterial and osteogenic properties plays a key role in “one‐stop‐shop” postoperative osteosarcoma management.

Selenium (Se) is an essential element for physiological bone homeostasis.^[^ ^29^, ^30^, ^31^, ^32^ ^]^ Se deficiency can lead to abnormal bone metabolism and trigger Kashin‐Beck disease (an endemic osteochondropathy), while exogenous Se supplement contributes to skeletal homeostasis and promotes bone regeneration following injuries.^[^ ^33^, ^34^, ^35^ ^]^ However, with a narrow therapeutic window, Se supplements can easily cause systemic toxicity, prompting the development of selenium nanoparticles (SeNPs) as a safer alternative. Of note, SeNPs can activate superoxide dismutase (SOD), transforming superoxide anion radical (·O_2_^−^) into H_2_O_2_, thereby elevating H_2_O_2_ levels within TME and promoting CDT efficiency.^[^ ^36^, ^37^, ^38^, ^39^, ^40^ ^]^ Besides, SeNPs are reported to possess anti‐bacterial properties against both gram‐positive and negative bacterium, as well as show great potential for refractory anti‐drug bacterial‐related infection treatment.^[^ ^41^, ^42^, ^43^ ^]^ Nonetheless, SeNPs exhibit low stability and are prone to micron‐scale aggregation, leading to compromised biocompatibility and bio‐reactivity.^[^ ^44^, ^45^, ^46^ ^]^ Layered double hydroxide (LDH) nanosheets, a representative 2D‐layered inorganic nanomaterial, have been proven to be promising nano‐agents for drug delivery with ultrathin layered structure and ultrahigh surface area.^[^ ^47^, ^48^, ^49^, ^50^, ^51^ ^]^ For example, MgAl‐LDH nanosheets were used as nanocontainers to effectively deliver chemical drugs.^[^ ^52^ ^]^ In addition, ultrathin CoFe‐LDH and CuFe‐LDH nanosheets could also act as Fenton or Fenton‐like agents to catalyze H_2_O_2_ into ·OH for high‐efficiency CDT.^[^ ^53^, ^54^ ^]^ Therefore, the incorporation and immobilization of SeNPs on the surface of LDH nanosheets rich in Fenton active can avoid the biotoxicity caused by SeNPs aggregation and enhance the CDT effect by promoting H_2_O_2_ generation based on SeNPs‐activated SOD activity. Importantly, 2D LDH nanosheets have also been reported to possess osteogenic properties by introducing bioactive magnesium (Mg^2+^), copper (Cu^2+^), iron (Fe^3+^), calcium (Ca^2+^), europium (Eu^3+^), and strontium (Sr^2+^) ions.^[^ ^55^, ^56^, ^57^, ^58^ ^]^ For instance, the incorporation of MgAl‐LDH microsheets into poly(methyl methacrylate) bone cement or the surface functionalization of MgAlEu‐LDH on porous hydroxyapatite scaffold boosted their bone formation efficiency and thus achieved superior bone repair.^[^ ^56^, ^57^ ^]^ Thus, the combination of LDH nanosheets and SeNPs holds the potential to synergistically promote bone defect repair and reconstruction.

**Figure 1:**
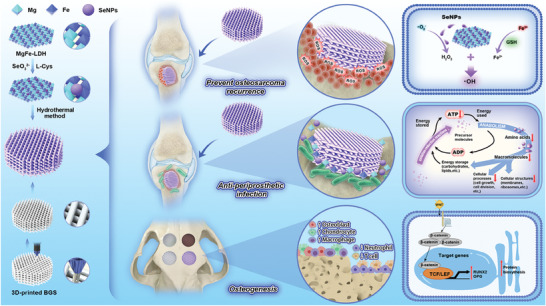
A multifunctional SeNPs‐incorporated MgFe‐LDH nanosheets platform for a “one‐stop‐shop” strategy for postoperative osteosarcoma eradication with antibacterial and bone reconstruction.

Herein, we synthesize MgFe‐LDH nanosheets (LDH) using a co‐precipitation approach, followed by the “in‐situ reduction method” to produce SeNPs‐incorporated LDH nanocomposite (LDH/Se). The LDH/Se is subsequently loaded onto a 3D‐printed bioactive glass scaffold (BGS) to obtain a multifunctional platform (BGS@LDH/Se) for osteosarcoma eradication with antibacterial and bone reconstruction (**Figure** **1**). The negatively charged SeNPs are homogeneously distributed on the LDH surface to prevent toxic aggregation and inactivation, which boosts SOD activation and ·O_2_^−^‐H_2_O_2_ conversion. Additionally, Fe^3+^ on MgFe‐LDH matrix is converted to Fe^2+^ by depleting glutathione (GSH) in the TME, which further triggers Fenton reaction and produces reactive oxygen species (ROS). In vivo experiments demonstrate that BGS@LDH/Se could completely prevent in‐situ tumor recurrence after osteosarcoma excision and avoid recurrence‐related death. Besides, the LDH/Se is proven to effectively halt drug‐resistant bacterial proliferation (>99%) by disturbing the fundamental energy and nutrient metabolism of bacteria. The incorporation of LDH/Se into BGS further eradicates drug‐resistant bacterial‐associated periprosthetic infection and subsequent osteomyelitis. Notably, the synergistic osteogenic properties of SeNPs as well as Mg^2+^ and Fe^3+^ on LDH matrix induce a 4.7‐folds and 2.0‐folds increment in bone mass compared with pristine BGS and LDH‐loaded BGS, respectively. Further transcriptome sequencing and single‐cell RNA‐sequencing reveal that the activated Wnt‐β‐catenin signal pathway as well as the increased osteoblasts, chondrocytes, and macrophages contribute to the exceptional osteogenic properties of BGS@LDH/Se. In conclusion, by developing a multifunctional BGS@LDH/Se platform with exceptional osteosarcoma eradication, antibacterial, and osteogenesis characteristics, we offer a promising strategy for postoperative “one‐stop shop” management of patients who need osteosarcoma resection and scaffold implantation.

## 2 Results and Discussion

**Figure 2:**
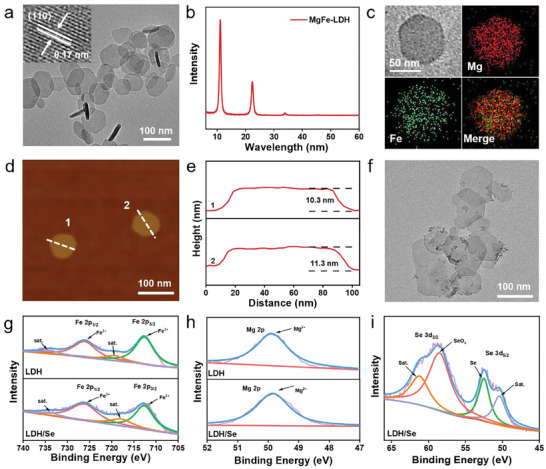
a) TEM images, b) XRD pattern, c) EDX mapping, d) AFM image, and e) corresponding height profiles of MgFe‐LDH nanosheets. f) TEM image of LDH/Se nanosheets. g) Fe 2p, h) Mg 2p, and (i) Se 3d XPS spectra of LDH/Se nanosheets.

The MgFe‐LDH nanosheets were prepared via a co‐precipitation method reported in a previous study.^[^ ^55^ ^]^ The MgFe‐LDH nanosheets were characterized by transmission electron microscope (TEM). As presented in **Figure** **2**a, MgFe‐LDH nanosheets display monodispersed hexagonal shape with a diameter size of 60–110 nm. The insert image in Figure 2a shows the lattice fringe space of 0.17 nm for (110) plane of LDHs, indicating the well‐reserved dominant crystal structure of MgFe‐LDH nanosheets. As displayed in X‐ray powder diffraction (XRD) pattern (Figure 2b), typical peaks are observed at 11.08° and 22.43°, attributed to the (003) and (006) planes of MgFe‐LDH, confirming its high crystallinity. The elemental composition of the MgFe‐LDH nanosheets was analyzed by energy dispersive X‐Ray spectroscopy (EDX) element mapping (Figure 2c), which confirms the presence of Mg and Fe through the entire nanosheet. Atomic force microscopy (AFM) height image reveals that the thickness of MgFe‐LDH nanosheets is 10.3−11.3 nm (Figure 2d,e).

The LDH/Se nanocomposite was then prepared by a facile in situ reduction method (see details in Methods). TEM image of LDH/Se nanosheets shows that well‐dispersed SeNPs are loaded on the surface of MgFe‐LDH without obvious change on the LDH nanosheets (Figure 2f). Subsequently, the chemical composition and valence state of the MgFe‐LDH and LDH/Se nanosheets were characterized by X‐ray photoelectron spectroscopy (XPS). The wide XPS spectra proved the presence of Mg and Fe in both MgFe‐LDH and LDH/Se nanosheets, with additional Se present in LDH/Se nanosheets (Figure S1, Supporting Information). In the Fe 2p XPS spectra (Figure 2g), peaks at 726.38 and 712.88 eV with satellite peaks at 733.98 and 719.48 eV correspond to Fe^3+^ 2p_1/2_ and 2p_3/2_, respectively, suggesting the existence of Fe^3+^ in MgFe‐LDH and LDH/Se nanosheets. Figure 2h shows the Mg 2p XPS spectra, where the peak at 49.38 eV is assigned to Mg^2+^ 2p, indicating the composition of Mg^2+^ species in MgFe‐LDH and LDH/Se nanosheets. In the Se 3d XPS spectrum of LDH/Se nanosheets (Figure 2i), the peak at 52.58 eV is assigned to Se(0), and the peak at 58.58 eV is assignable to Se(IV), which may be due to the slight oxidation of SeNPs in air.

**Figure 3:**
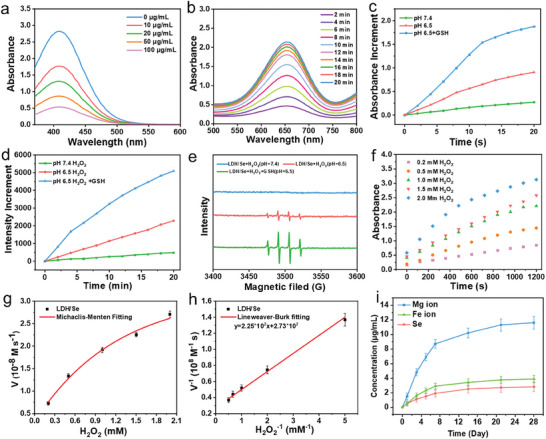
a) GSH depletion by different concentrations of LDH/Se (0, 10, 20, 50, and 100 µg mL^−1^). b) The UV absorbance spectra of TMB in the presence of LDH/Se, H_2_O_2_, and GSH at pH 6.5. c) The absorbance of ox‐TMB at 652 nm in the presence of LDH/Se under different conditions: H_2_O_2_ (pH 7.4), H_2_O_2_ (pH 6.5), and H_2_O_2_ + GSH (pH 6.5). d) The fluorescence intensity of TA at 420 nm in the presence of LDH/Se under different conditions: H_2_O_2_ (pH 7.4), H_2_O_2_ (pH 6.5), and H_2_O_2_ + GSH (pH 6.5). e) ESR spectra of DMPO/·OH for the LDH/Se nanosheets under different conditions: H_2_O_2_ (pH 7.4), H_2_O_2_ (pH 6.5), and H_2_O_2_ + GSH (pH 6.5). f) The absorbance of ox‐TMB at 652 nm in the presence of LDH/Se and different concentrations of H_2_O_2_. g) Michaelis‐Menten fitting and h) Lineweaver–Burk fitting of LDH/Se against H_2_O_2_ concentration. i) Mg^2+^, Fe^3+^ and Se release from BGS@LDH/Se.

Based on the presence of Fe^3+^ in LDH/Se nanosheets and overexpressed GSH in the TME, the regulating ability of LDH/Se toward GSH depletion was investigated by 5,5‐dithiobis‐(2‐nitrobenzoic acid) (DTNB) assay. As presented in **Figure** **3**a, the absorbance of DTNB decreased with the increase of LDH/Se concentration from 0–100 µg mL^−1^, demonstrating the excellent GSH consumption ability of LDH/Se. To further verify the reaction between LDH/Se and GSH, XPS was carried out to explore the change in the valence state of Fe in LDH/Se after reaction. As observed in the XPS spectra (Figure S2, Supporting Information), new peaks at 723.08 and 711.88 eV assigned to Fe^2+^ 2p_1/2_ and 2p_3/2_ were found in LDH/Se after reacting with GSH, indicating that partial Fe^3+^ was reduced to Fe^2+^ that could catalyze H_2_O_2_ into ·OH based on Fenton reaction. The above‐mentioned results show that LDH/Se is expected to regulate the GSH level in the TME and strengthen the CDT performance by preventing GSH‐induced ROS clearance.

The OH generation through the Fenton reaction was first investigated via 3,3′,5,5′‐tetramethylbenzidine (TMB) assay. As presented in Figure 3b,c and Figure S3 (Supporting Information), LDH/Se in a buffer solution containing H_2_O_2_ at pH 7.4 (simulated normal tissue environment) executed a negligible influence on TMB oxidation, while obvious time‐dependent TMB oxidation was found in a buffer solution containing H_2_O_2_ at pH 6.5 (simulated weakly acidic TME), indicating the pH‐responsive CDT performance of LDH/Se. Moreover, TMB oxidation was further enhanced after the addition of GSH, implying the more pronounced ·OH‐generating activity of LDH/Se in the TME. Terephthalic acid (TA) probe was also utilized to monitor the ·OH generation of LDH/Se at different conditions. In Figure 3d and Figure S4 (Supporting Information), the fluorescence intensity of TA solution at 420 nm did not change obviously at pH 7.4, but significantly increased over time at pH 6.5. Expectedly, the fluorescence intensity was further enhanced in the presence of GSH, confirming the TME‐responsive CDT performance of LDH/Se. Such a result was further verified by electron spin resonance (ESR) spectroscopy using 5,5‐dimethyl‐1‐pyrroline N‐oxide (DMPO) probe,^[^ ^59^ ^]^ as the strongest characteristic (1:2:2:1) ·OH signal was observed at pH 6.5 in the existence of GSH (Figure 3e). Subsequently, the catalytic activity of LDH/Se with H_2_O_2_ as the substrate was evaluated using typical enzyme‐kinetics theory (Figure 3f–h). Through the Beer–Lambert law and Michaelis–Menten curve, the Michaelis constant (*K*_m_) and maximal reaction rate (*V*_max_) of LDH/Se calculated from the Lineweaver–Burk plot were 0.82 mm and 3.67 × 10^−8^ M s^−1^, respectively, guaranteeing a mild and stable catalytic Fenton reaction of LDH/Se in the TME, which is expected to induce excellent anti‐cancer therapeutic effect.

Based on the above results, LDH/Se nanosheets were loaded on 3D‐printed BGS to prepare BGS@LDH/Se composite scaffolds. As shown in the digital photos (Figure S5, Supporting Information), compared with the pristine BGS (white), the surface of the BGS@LDH/Se scaffold became dark yellow, suggesting the complete cover of BGS by LDH/Se nanosheets. Then, the surface micro‐morphology of BGS@LDH/Se was characterized by scanning electron microscopy (SEM). In Figure S6 (Supporting Information), a smooth surface structure was found in the pristine BGS scaffold, while a relatively rough surface was observed in the BGS@LDH/Se scaffold, which is conducive to cell adhesion. According to the energy dispersive spectroscopic (EDS) elemental mapping images, Mg, Fe, Se (from LDH/Se), Si, P, O, and Ca (from BGS) elements were uniformly distributed on the surface of BGS@LDH/Se (Figure S7, Supporting Information), confirming the successful functionalization of BGS. The mechanical properties of scaffolds before and after functionalization of LDH/Se were investigated by electronic universal testing machine. As shown in Figure S8 (Supporting Information), the compression modulus and compressive strength of BGS@LDH/Se were 2.2 times and 1.3 times that of the BGS, indicating that LDH/Se functionalization improved the compressive capacity of BGS scaffolds. In addition, the release of Mg^2+^, Fe^3+^, and Se from BGS@LDH/Se in PBS was characterized by inductively coupled plasma‐atomic emission spectroscopy (ICP‐AES). As presented in Figure 3i, LDH/Se nanosheets could sustainably release bioactive Mg^2+^, Fe^3+^, and Se within 28 days, which are beneficial for promoting bone regeneration.

**Figure 4:**
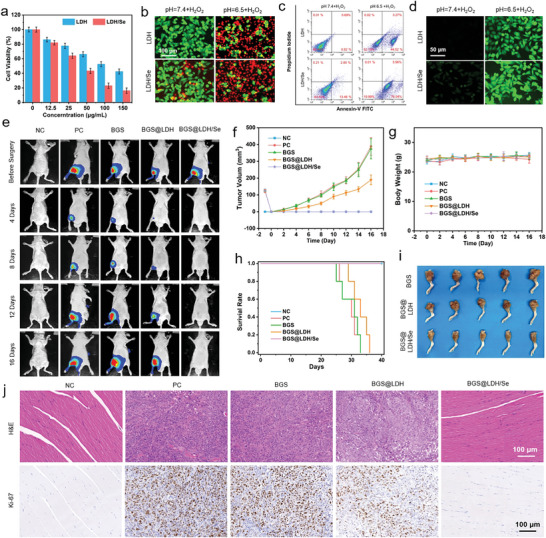
a) MTT experiments evaluating the CDT efficiency of LDH and LDH/Se against Saos‐2 cells. b) Calcein‐AM/PI double staining evaluating CDT efficiency of LDH and LDH/Se under normal and acidulous microenvironment. c) Flow cytometry analysis accessing the CDT efficiency of LDH and LDH/Se against Saos‐2 cells. d) DCFH‐DA fluorescent staining of Saos‐2 cells treated with LDH and LDH/Se under different pH environments. e) Bioluminescence small animal imaging monitoring the osteosarcoma recurrence and progression in NC, PC, BGS, BGS@LDH, and BGS@LDH/Se groups up to 16 days. f) Tumor volume curve of osteosarcoma‐bearing mice in NC, PC, BGS, BGS@LDH, and BGS@LDH/Se groups. g) Body weight of osteosarcoma‐bearing mice in NC, PC, BGS, BGS@LDH, and BGS@LDH/Se groups at certain time points. h) Forty‐day survivorship curve of osteosarcoma‐bearing mice in NC, PC, BGS, BGS@LDH, and BGS@LDH/Se groups. i) Gross image of the knee and surrounding tissues of osteosarcoma‐bearing mice in BGS, BGS@LDH, and BGS@LDH/Se groups. j) H&E and Ki‐67‐stained tumor tissue slices of osteosarcoma‐bearing mice in NC, PC, BGS, BGS@LDH, and BGS@LDH/Se groups.

The aforementioned results fully demonstrate the satisfactory CDT‐mediated antitumor performance and potential LDH‐induced osteogenic activity of LDH/Se nanosheets. In view of this, the biocompatibility of LDH, Na_2_SeO_3_, and LDH/Se was evaluated using methyl thiazolyl tetrazolium (MTT) method in Saos‐2 cells (human osteosarcoma cells). As shown in Figure S9 (Supporting Information), Saos‐2 cells co‐cultured with LDH presented great viability, while insufficient biocompatibility of Na_2_SeO_3_ was observed in both neutral (pH 7.4) and slightly acidic (pH 6.5) environments in relatively high concentrations. Interestingly, the cell viability of LDH/Se was significantly higher compared with pristine Na_2_SeO_3_, suggesting that LDH facilitated the uniform dispersion of Se element and avoided the formation of toxic selenium clusters. Subsequently, the Saos‐2 cell‐killing efficiency of the LDH and LDH/Se was evaluated. In **Figure** **4**a, the cell viability of the LDH/Se group was measured to be 16.2% upon 150 µg mL^−1^ LDH/Se in the presence of H_2_O_2_ at pH 6.5, much lower than that of the LDH group, indicating that SeNPs could significantly enhance the CDT efficiency of LDH, which is attributed to the SeNPs‐activated SOD activity to transform ·O_2_^−^ into H_2_O_2_.

The CDT efficiency of LDH/Se was further confirmed by calcein acetoxymethyl ester and propidium iodide (Calcein‐AM/PI) double staining assays, where the strongest red fluorescence (PI) indicating cell death was observed only in the LDH/Se + H_2_O_2_ group under slightly acidic (pH 6.5) environment (Figure 4b; Figure S10, Supporting Information). Flow cytometry analysis also revealed that LDH/Se could effectively induce extensive Saos‐2 apoptosis (Figure 4c). In addition, DCFH‐DA was adopted as a fluorescent ROS indicator to examine intracellular ROS levels, of which the LDH/Se + H_2_O_2_ group exhibited the strongest fluorescence intensity (Figure 4d), indicating abundant ROS production. Western blot analysis further revealed that LDH/Se could significantly induce upregulation of superoxide dismutase 1 and downregulation of phosphorylated signal transducer and transcription activator 3 protein expression (Figure S11, Supporting Information), indicating the aggravating oxidative damage of Saos‐2 cells triggered by LDH/Se.

Inspired by the encouraging in vitro results, the efficacy of LDH/Se in treating in situ bone tumors was evaluated in a tibial osteosarcoma model of nude mice. The tibial osteosarcoma was allowed to grow in situ for 14 days before resection surgery followed by BGS, BGS@LDH, or BGS@LDH/Se implantation into the surgical defects. For clarity, we included both a negative control group (NC), which did not receive initial subperiosteal osteosarcoma cell injection but underwent tibial bone defect modeling with no scaffold implantation, and a positive control group (PC), which underwent initial subperiosteal osteosarcoma cell injection followed by osteosarcoma resection after 2 weeks without scaffold implantation. Bioluminescence small animal imaging revealed complete inhibition of osteosarcoma recurrence in BGS@LDH/Se group, similar to the outcome of the NC group, suggesting the effective elimination of osteosarcoma by BGS@LDH/Se. By contrast, rapid osteosarcoma recurrence and deterioration occurred similarly in the PC and BGS groups. The BGS@LDH group also exhibited recurrence but with a delayed deterioration ratio, indicating moderate yet insufficient anti‐osteosarcoma properties (Figure 4e). Similar results were also confirmed in tumor volume quantification, where the tumor volume in BGS@LDH group was significantly smaller than that in PC and BGS groups, while no tumor was detected in NC and BGS@LDH/Se groups up to the end monitor point (Day 16), suggesting that the deficient tumor‐recurrence prophylactic ability of LDH could be significantly strengthened by SeNPs (Figure 4f). During the monitoring period, no significant change in the body weight of mice was observed in any group, indicating negligible systematic toxicity of BGS@LDH and BGS@LDH/Se (Figure 4g). Moreover, the NC and BGS@LDH/Se groups presented significantly improved survival rates compared with the PC, BGS, and BGS@LDH groups, with none of the mice dying for up to 40 days (Figure 4h). Correspondingly, normal musculature and regular knee joint were observed in NC and BGS@LDH/Se groups but a mass of osteosarcoma tissue invading the knee joint as well as adjacent soft tissue was exhibited in the PC and BGS groups 16th day postoperatively, whereas BGS@LDH group demonstrated an intermediate osteosarcoma volume and invasion degree between BGS and BGS@LDH/Se groups (Figure 4i; Figure S12, Supporting Information). Hematoxylin and eosin (H&E) staining and Ki‐67 staining of tumor slices revealed numerous metachromatic tumor cells and active malignant proliferative behavior in the PC, BGS, and BGS@LDH groups (Figure 4j). By contrast, normal peri‐articular muscle tissues were detected in the NC and BGS@LDH/Se groups (Figure 4j), demonstrating the excellent in vivo CDT performance of BGS@LDH/Se for preventing osteosarcoma recurrence.

**Figure 5:**
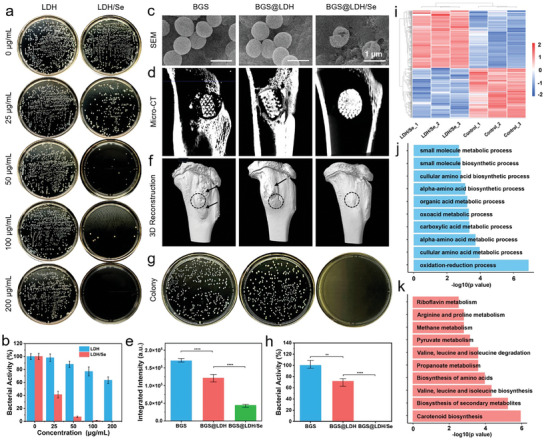
a) Digital images of bacterial colonies formed by *MRSA* incubated with LDH or LDH/Se at different concentrations. b) Quantitative analysis of the *MRSA* colonies pretreated with LDH or LDH/Se at indicated concentrations. c) SEM images of *MRSA* inoculated on BGS, BGS@LDH, and BGS@LDH/Se. d) Micro‐CT images indicating the severity of periprosthetic infection treated with indicated therapies in rabbit upper tibia. e) Quantitative analysis of the abnormal integrated intensity of Micro‐CT in BGS, BGS@LDH, and BGS@LDH/Se groups. f) 3D reconstruction of the Micro‐CT imaging evaluating infection‐responsive hyperosteogeny in BGS, BGS@LDH, and BGS@LDH/Se groups. g) Digital images of colonies incubated from the adjacent muscle tissue around BGS, BGS@LDH, and BGS@LDH/Se. h) Quantitative analysis of the bacterial colonies formed by the periarticular tissues from BGS, BGS@LDH, and BGS@LDH/Se groups. i) Heatmap depicting the differential gene expression paradigm between LDH/Se group and control group. j) GO and k) KEGG analysis enriching the downregulated genes into essential biological processes. Data are expressed as mean ± S.D. (*n* = 3). Statistical comparisons were made by one‐way ANOVA (for multiple comparisons): ^**^ *p* < 0.01, ^****^ *p* < 0.0001.

Periprosthetic infection is a serious complication for implant‐involved surgery, especially for weakened osteosarcoma patients who bear impaired tolerance to revision surgery. Drug‐resistant bacteria‐associated infections exhibit stubborn resistance to regular antibiotics, further complicating the situation. Herein, the in vitro drug‐resistant bacteria elimination performance of LDH and LDH/Se was evaluated using *Methicillin‐resistant Staphylococcus aureus* (*MRSA*, a typical drug‐resistant bacteria, gram‐positive) and *Escherichia coli* (*E. coli*, gram‐negative). Specifically, *MRSA* or *E. coli* was co‐incubated with different concentrations of LDH and LDH/Se (0, 25, 50, 100, and 200 µg mL^−1^) and propagated on agar plates for colony formation.^[^ ^60^, ^61^ ^]^ As shown in **Figures** **5**a and S13a (Supporting Information), a slight anti‐bacterial effect was observed with increased LDH concentration, which may be attributed to the antibacterial properties of Mg^2+^ and Fe^3+^ released from LDH. Notably, a significant reduction in bacterial colonies was detected in the LDH/Se group, where complete bacteria elimination was obtained at the concentration of 200 µg mL^−1^ (Figure 5b; Figure S13b, Supporting Information), highlighting the effective antibacterial efficiency of SeNPs for both gram‐positive and negative bacteria. Moreover, to visually verify the antibacterial efficacy of LDH/Se, *MRSA* or *E. coli* was directly seeded on the surface of BGS@LDH/Se and detected under SEM,^[^ ^62^ ^]^ where a shrunken and cracked appearance of bacteria in the BGS@LDH/Se group while intact bacteria morphology in the BGS and BGS@LDH groups were observed (Figure 5c; Figure S14, Supporting Information).

Encouraged by the promising in vitro antibacterial performance, the in vivo drug‐resistance bacteria elimination efficiency of BGS@LDH/Se was investigated in New Zealand white rabbit periprosthetic infection model. The BGS, BGS@LDH, or BGS@LDH/Se scaffolds were immersed in *MRSA* suspension and then implanted into a bone defect on the upper end of the tibia to simulate postoperative periprosthetic infection. 3D reconstructed Micro‐computed tomography (Micro‐CT) imaging of the tibia was performed 1 month after scaffold implantation, which revealed evident osteomyelitis in the BGS and BGS@LDH groups, while the BGS@LDH/Se group displayed a clear normal view (Figure 5d). Further quantitative analysis confirmed significantly reduced abnormal integrated intensity in the BGS@LDH/Se group compared with that in BGS and BGS@LDH groups (Figure 5e). 3D reconstruction of the Micro‐CT imaging demonstrated an infection‐responsive pathological hyperosteogeny in BGS and BGS@LDH groups but not BGS@LDH/Se group (Figure 5f). Additionally, the adjacent muscle tissue around the scaffold was harvested, ground, and subjected to LB agar inoculation, of which amounts of colonies were formed in the BGS and BGS@LDH groups, while no colony formation was observed in the BGS@LDH/Se group (Figure 5g,h), highlighting the potential of BGS@LDH/Se for periprosthetic infection prevention.

Despite great antibacterial efficiency, the underlying mechanism of the intrinsic antibacterial properties of LDH/Se was unclear, for which the RNA sequence for *MRSA* co‐cultured with either regular medium or medium containing LDH/Se (100 µg mL^−1^) was performed. As shown in Figure 5i, different gene expression patterns were clustered in the LDH/Se and control groups, respectively. A total of 885 differentially expressed genes (P < 0.05&|log2 Fold Change| > 1) were identified, of which 475 were upregulated genes (marked in red) and 410 were downregulated genes (marked in green) (Figure S15, Supporting Information). To identify altered biological processes in *MRSA* co‐cultured with LDH/Se, Gene Ontology (GO) analysis was involved. As depicted in Figure 5j, the top 10 altered biological processes are primarily associated with the respiratory electron transport processes and amino acid biosynthesis and metabolic processes, which play essential roles in bacterial survival. Importantly, “oxidation‐reduction processes” is the top one physiological process inhibited by BGS@LDH/Se. In fact, oxidation‐reduction processes are pivotal for bacterial energy generation by driving ATP production. Nutrient metabolism, including organic matter breakdown and nitrogen fixation, is also facilitated by these processes. Notably, inhibition of oxidation‐reduction processes can reduce ROS neutralization and exacerbate oxidative damage in bacteria.^[^ ^63^, ^64^ ^]^ Besides, the Kyoto Encyclopedia of Genes and Genomes (KEGG) analysis further revealed that the metabolism of carotenoid, secondary metabolic, and various amino acids were disturbed by LDH/Se (Figure 5k). It's noteworthy that carotenoid biosynthesis holds potent antioxidant properties, scavenging ROS and shielding bacterial cells from oxidative stress.^[^ ^65^ ^]^ Moreover, carotenoids can bolster the stability and integrity of bacterial cell membranes, which is vital for bacterial survival and vitality.^[^ ^66^ ^]^ These results illustrate that the LDH/Se can inhibit bacterial activity and proliferation by impeding the fundamental energy and nutrient metabolism of bacteria.

**Figure 6:**
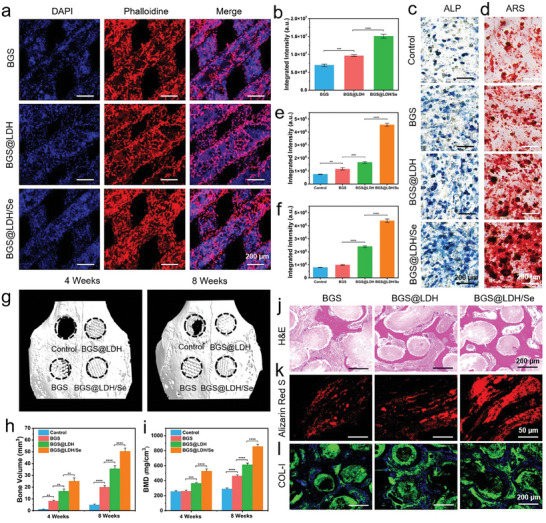
a) CLSM images of hBMSCs stained with DAPI (displaying blue fluorescence) and rhodamine phalloidin (displaying red fluorescence) adhered on BGS, BGS@LDH, and BGS@LDH/Se. b) Quantitative analysis of the integrated intensity of adhered hBMSCs on the surface of BGS, BGS@LDH, and BGS@LDH/Se. Optical microscope images of c) ALP staining and d) ARS staining of hBMSCs in control group or co‐cultured with BGS, BGS@LDH, and BGS@LDH/Se. Quantitative analysis of the integrated intensity of e) ALP and f) ARS in the control, BGS, BGS@LDH, and BGS@LDH/Se groups. g) Front view of the 3D reconstructed Micro‐CT images exhibiting new bone formation induced by BGS, BGS@LDH, or BGS@LDH/Se 4 and 8 weeks postoperatively. Quantitative analysis of the h) regenerated bone volume and i) bone mineral density in control, BGS, BGS@LDH, and BGS@LDH/Se groups based on the 3D reconstructed Micro‐CT images. j) H&E staining of new bone formation induced by BGS, BGS@LDH, and BGS@LDH/Se. k) CLSM images of calcium deposition labeled with ARS in BGS, BGS@LDH, and BGS@LDH/Se groups. l) Immunofluorescence images revealing COL‐I expression around in BGS, BGS@LDH, and BGS@LDH/Se groups. Data are expressed as mean ± S.D. (*n* = 3). Statistical comparisons were made by one‐way ANOVA (for multiple comparisons): ^**^ *p* < 0.01, ^***^ *p* < 0.001, ^****^ *p* < 0.0001.

To investigate the osteogenic properties of BGS, BGS@LDH, and BGS@LDH/Se, the biocompatibility of the scaffolds for human bone marrow mesenchymal stem cells (hBMSCs) was first accessed. Live/Dead staining experiments were conducted on hBMSCs incubated with various scaffolds. The results showed abundant live cells and negligible dead cells in BGS, BGS@LDH, and BGS@LDH/Se groups during the observation period (7 days), similar to the negative control group (incubated with regular cell culture medium) (Figure S16, Supporting Information). Moreover, CCK‐8 assays confirmed the great biocompatibility of BGS, BGS@LDH, and BGS@LDH/Se scaffolds for hBMSCs over 7 days, as indicated by similar CCK‐8 values across the control, BGS, BGS@LDH, and BGS@LDH/Se groups. Moreover, the BGS@LDH and BGS@LDH/Se groups had higher CCK‐8 values compared with the control and BGS groups across the observation period, with the BGS@LDH/Se group showing the highest values, suggesting the proliferation‐provoking properties of Mg^2+^, Fe^3+^, and SeNPs of BGS@LDH/Se (Figure S17, Supporting Information). In addition, further hemolysis test revealed negligible hemolysis in rabbit venous blood incubated with the leachates of BGS, BGS@LDH, and BGS@LDH/Se scaffolds, resembling the results of negative control group (incubated with PBS) (Figure S18, Supporting Information), while significant hemolysis was observed in the positive control group (incubated with deionized water), confirming the good biocompatibility of the involved scaffolds. To evaluate the cellular adhesion performance of the scaffolds, hBMSCs were directly seeded on BGS, BGS@LDH, or BGS@LDH/Se. Significantly denser hBMSCs were observed on BGS@LDH/Se surface compared with BGS (2.2‐folds) and BGS@LDH (1.6‐folds), indicating the superiority of BGS@LDH/Se in promoting cell adhesion (**Figure** **6**a,b). Subsequently, alkaline phosphatase (ALP) staining and Alizarin red S (ARS) assays were employed to investigate the osteogenic properties of the scaffolds. Fourteen days after co‐culture, remarkably higher ALP activities and enhanced calcium deposition were observed in BGS@LDH and BGS@LDH/Se groups compared with the control and BGS groups (Figure 6c,d). Notably, the BGS@LDH/Se group exhibited the highest ALP and ARS activity (Figure 6c,d). Further quantitative analysis demonstrated 6.1‐folds and 2.8‐folds integrated intensity increment of ALP, as well as 5.5‐folds and 1.8‐folds of ARS in BGS@LDH/Se group compared with that of the control group and BGS@LDH group (Figure 6e,f), respectively, indicating the synergistic effects of SeNPs and LDH for enhanced osteogenic performance.

Encouraged by the excellent in vitro biocompatibility and osteogenic properties, the in vivo bone regeneration abilities of the BGS@LDH/Se were investigated. The calvarial critical‐sized defect models were established in New Zealand white rabbits followed by different scaffold implantation. Four and eight weeks after surgery, the skulls were harvested and underwent 3D reconstructed Micro‐CT scanning. As presented in Figure 6g and Figure S19 (Supporting Information), significantly enhanced new bone formation was found in BGS@LDH/Se group compared with BGS and BGS@LDH groups at both 4 and 8 weeks postoperatively, while BGS@LDH also induced more bone formation compared with pristine BGS. Of note, abundant bone regeneration across the defect area and complete skull reconstruction were obtained in BGS@LDH/Se group 8 weeks after surgery (Figure 6g; Figure S19, Supporting Information). Quantitative analysis of 3D reconstructed Micro‐CT images revealed 2.5‐folds and 1.4‐folds bone volume, 1.8‐folds and 1.4‐folds bone mineral density (BMD), and 4.7‐folds and 2.0‐folds total bone mass increment in BGS@LDH/Se group compared with BGS and BGS@LDH groups (Figure 6h,i; Figure S20, Supporting Information), respectively. H&E and Sirius red staining also verify the enhanced bone regeneration induced by BGS@LDH/Se, where remarkably more bone formation was observed around the BGS@LDH/Se than that around BGS and BGS@LDH groups (Figure 6j; Figure S21, Supporting Information). Furthermore, ARS was intraperitoneally injected into modeled rabbits 4 and 6 weeks after scaffold implantation to label neo‐biomineralization. As shown in Figure 6k, significantly more biomineralization deposition and larger calcium nodules were detected around the scaffolds in the BGS@LDH/Se group 8 weeks after surgery. In addition, type I collagen (COL‐I) as the main component of bone tissue was also significantly up‐regulated in BGS@LDH/Se group compared with that in BGS and BGS@LDH 8 weeks postoperatively (Figure 6l). Of note, the circular, intensely stained green areas here are threads of 3D‐printed BGS oriented perpendicular to the cutting section, while specific COL‐I staining is indicated by the green fluorescence surrounding these circular stained regions, which are co‐localized with DAPI dispersal. Moreover, to evaluate the systematic biosafety of the BGS, BGS@LDH, and BGS@LDH/Se scaffolds, histological analyses were performed on the heart, liver, spleen, lung, and kidney tissues of rabbits in control group (normal rabbits without skull defects) and experimental group (four circular defects created in the rabbit skulls were filled with BGS, BGS@LDH, and BGS@LDH/Se) at 4 and 8 weeks post‐implantation. As expected, no significant histological differences were observed in any organs between the control group and the experimental group, demonstrating the excellent in vivo biocompatibility of the scaffolds (Figure S22, Supporting Information).

**Figure 7:**
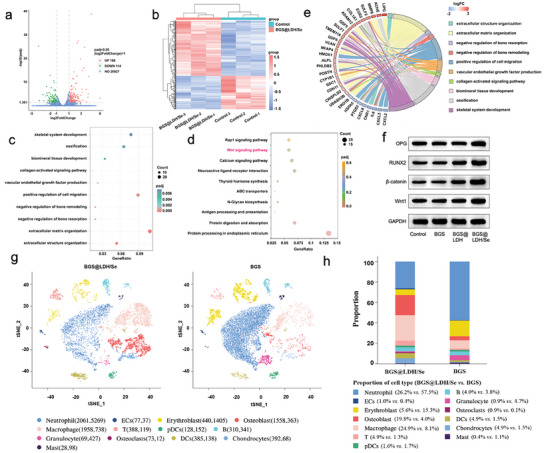
a) The heatmap and b) volcano plot exhibiting the differentially expressed genes. Cutoff: P value < 0.05 and |log2 FC| > 1. c) Top 10 osteogenic biological processes enriched by differentially expressed genes using GO analysis. d) Top 10 signal pathways enriched by differentially expressed genes using KEGG analysis. e) Chord diagram visualizing the correlation between the top 10 osteogenic biological processes and the top 30 differentially expressed osteogenic genes ranked with FoldChange. f) Western blot accessing the expression of proteins involved in Wnt signal pathway (Wnt1, β‐catenin, RUNX2, and OPG) of hBMSCs in control group or co‐cultured with BGS, BGS@LDH, and BGS@LDH/Se. g) t‐distributed Stochastic Neighbor Embedding (t‐SNE) visualizing cell cluster around BGS@LDH/Se and BGS in reduced dimensions. h) The proportion of various cellular clusters in the indicated group. ECs, Endothelial cells; pDCs, plasmacytoid dendritic cells; DCs, dendritic cells.

To explore the underlying mechanism contributing to the superior osteogenic performance of BGS@LDH/Se, RNA transcriptome sequencing was performed. The volcano plot and heatmap depicted differentially expressed genes of hBMSCs cultured with regular medium or BGS@LDH/Se, of which actively expressed genes were marked in red and silenced genes were marked in green (**Figure** **7**a,b). A total of 302 differentially expressed genes (P < 0.05&|log2 Fold Change| > 1) were identified, containing 188 upregulated genes and 114 downregulated genes. The top 10 osteogenic biological processes with significant statistical difference were determined utilizing GO analysis by enriching differentially expressed genes (Figure 7c), which suggests that the exceptional osteogenic properties of BGS@LDH/Se were attributed to its ability in promoting extracellular matrix organization and biomineral tissue development, inhibiting bone resorption, as well as activating collagen signaling pathway. Of note, the extracellular matrix and extracellular structural organization are the most upregulated biological processes, which play a pivotal role in facilitating subsequent osteogenesis by establishing a supportive matrix platform.^[^ ^67^, ^68^ ^]^ To further identify the specific signal pathway engaging the osteogenesis processes of BGS@LDH/Se, KEGG analysis was conducted. As shown in Figure 7d, the Wnt signal pathway that typically participates in bone formation processes was identified to be significantly activated in BGS@LDH/Se group than that in control group. Moreover, to further identify differentially expressed gene sets mediated by BGS@LDH/Se, Gene Set Enrichment Analysis (GSEA) was performed with the GSEA software (http://software.broadinstitute.org/gsea/index.jsp). Interestingly, GSEA also revealed that the gene set of the Wnt signaling pathway was positively correlated with BGS@LDH/Se intervention (Figure S23, Supporting Information), further confirming the essential role of the Wnt signaling pathway in BGS@LDH/Se‐mediated enhanced osteogenesis. Furthermore, the top 10 osteogenic biological processes enriched by GO analysis and the top 30 differentially expressed genes related to these processes (ranked by FoldChange) were orchestrated in a chord diagram for visualization (Figure 7e). It has been widely reported that the downstream osteogenic promoters of Wnt signal pathway were β‐catenin/RUNX2 and β‐catenin/OPG, thus the Wnt1, β‐catenin, RUNX2, and OPG gene expression levels of hBMSCs were detected using quantitative polymerase chain reaction (qPCR) assays. As illustrated in Figure S24 (Supporting Information), the highest transcription levels of Wnt1, β‐catenin, RUNX2, and OPG were detected in the BGS@LDH/Se group compared with other groups. Although transcriptome sequencing results suggested that the Wnt signaling pathways may contribute to the osteogenic properties of BGS@LDH/Se, gene expression alternations do not necessarily translate to protein expression or physiological function changes. Thus, Western blot assays were performed to confirm the participation of Wnt signal pathway in BGS@LDH/Se‐mediated osteogenesis. As shown in Figure 7f, remarkably upregulated Wnt1, β‐catenin, RUNX2, and OPG expression was observed in BGS@LDH/Se compared with other groups. Quantitative analysis of protein bands showed 1.8‐folds, 2.4‐folds, 2.0‐folds, and 1.8‐folds increments of Wnt1, β‐catenin, RUNX2, and OPG in BGS@LDH/Se group compared with that in control group (Figure S25, Supporting Information), respectively. Besides, immunofluorescence staining of Wnt signaling pathway‐associated biomarkers was conducted in prepared rabbit skull slides from the control, BGS, BGS@LDH, and BGS@LDH/Se areas. As shown in Figure S26 (Supporting Information), only sporadic positive staining of Wnt1, β‐catenin, RUNX2, and OPG was observed in the control and BGS groups. In contrast, significantly more positive staining of these markers was detected in the BGS@LDH/Se group, confirming the participation of Wnt signal pathway in the osteogenic processes of BGS@LDH/Se. A slightly enhanced RUNX2 immunofluorescence staining was observed in the BGS@LDH group compared with control and BGS groups, but still significantly weaker than that in the BGS@LDH/Se group. Thus, different analytical methodologies of the RNA‐sequencing data, in vitro transcriptional and translational verification, and in vivo immunofluorescence staining comprehensively demonstrated that the Wnt signaling pathway plays a crucial role in mediating the superior osteogenic properties of BGS@LDH/Se.

Besides the alternation in gene and signal pathway level, the in vivo cell cluster changes around the BGS@LDH/Se were explored by single‐cell RNA‐sequencing 2 weeks after scaffold implantation. Consequently, a total of 17034 cells were identified, with 9167 in the BGS group and 7867 in the BGS@LDH/Se group, from which 13 cell subgroups were annotated. As shown in Figure 7g,h, significantly more osteoblasts (1558 vs 363) and chondrocytes (392 vs 68) contributing to bone formation were identified in the BGS@LDH/Se group compared with that in BGS group. In addition, an obviously higher macrophage number (1958 vs 738) was annotated in the BGS@LDH/Se group (Figure 7g,h), which was considered to play an essential role in constructing early‐phase osteogenic inflammatory microenvironment after bone injuries. Furthermore, remarkably fewer neutrophils (2061 vs 5269) were found in BGS@LDH/Se group compared with BGS group (Figure 7g,h). As neutrophils play a crucial role in enhancing innate immune responses and promoting rejection reactions,^[^ ^69^ ^]^ the increase in neutrophil count in the BGS group compared with BGS@LDH/Se may indicate a more intense rejection response following scaffold implantation, suggesting inferior biocompatibility of BGS relative to BGS@LDH/Se. In conclusion, single‐cell sequencing results further elucidated the osteogenic mechanism of BGS@LDH/Se in cell dimension.

## 3 Conclusion

In conclusion, we developed a versatile scaffold by incorporating SeNPs‐incorporated MgFe‐LDH onto a 3D‐printed BGS. The negatively charged SeNPs were uniformly dispersed on the surface of MgFe‐LDH to mitigate toxic aggregation and inactivation. This arrangement enhanced the activation of SOD and the conversion of ·O^2−^ to H_2_O_2_. Furthermore, within the MgFe‐LDH matrix, the presence of Fe^3+^ was converted to Fe^2+^ through GSH depletion, which subsequently initiated the Fenton reaction, leading to the generation of ROS and tumor cell killing. Besides, LDH/Se has been demonstrated to effectively impede the proliferation of drug‐resistant bacteria (>99%) by disrupting fundamental energy and nutrient metabolism in bacteria. The integration of LDH/Se into BGS not only eliminated drug‐resistant bacterial‐related periprosthetic infections but also prevented subsequent osteomyelitis. Notably, the synergistic osteogenic effects of SeNPs, as well as Mg^2+^ and Fe^3+^ on the LDH matrix, resulted in an exceptional increase in bone mass compared to pristine BGS. Further transcriptome sequencing and single‐cell RNA‐sequencing analyses revealed that the Wnt‐β‐catenin signaling pathway, along with increased osteoblasts, chondrocytes, and macrophages, contributed to the enhanced osteogenic properties of BGS@LDH/Se. In conclusion, by developing a multifunctional BGS@LDH/Se platform with outstanding CDT efficiency, intrinsic antibacterial abilities, and enhanced osteogenic properties, we proposed a promising strategy for postoperative “one‐stop‐shop” management of osteosarcoma.

## Conflict of Interest

The authors declare no conflict of interest.

## Supporting information

Supporting Information

## Data Availability

The data that support the findings of this study are available from the corresponding author upon reasonable request.

## References

[1] C. A. Arndt , W. M. Crist , N. Engl. J. Med. 1999, 341, 342. 10423470 10.1056/NEJM199907293410507

[2] H. C. Beird , S. S. Bielack , A. M. Flanagan , J. Gill , D. Heymann , K. A. Janeway , J. A. Livingston , R. D. Roberts , S. J. Strauss , R. Gorlick , Nat. Rev. Dis. Primers 2022, 8, 77. 36481668 10.1038/s41572-022-00409-y

[3] J. Ritter , S. S. Bielack , Ann. Oncol. 2010, 21, vii320. 20943636 10.1093/annonc/mdq276

[4] R. J. Grimer , Lancet Oncol. 2005, 6, 85. 15683817 10.1016/S1470-2045(05)01734-1

[5] H. A. Finn , M. A. Simon , Clin. Orthop. Relat. Res. 1991, 262, 108. 1984905

[6] M. A. Simon , J. Bone Joint Surg. Am. 1988, 70, 307. 3277972

[7] M. S. Kim , S. Y. Lee , T. R. Lee , W. H. Cho , W. S. Song , J. S. Koh , J. A. Lee , J. Y. Yoo , D. G. Jeon , Ann. Oncol. 2009, 20, 955. 19153123 10.1093/annonc/mdn723

[8] S. S. Bielack , B. Kempf‐Bielack , G. Delling , G. U. Exner , S. Flege , K. Helmke , R. Kotz , M. Salzer‐Kuntschik , M. Werner , W. Winkelmann , A. Zoubek , H. Jürgens , K. Winkler , J. Clin. Oncol. 2002, 20, 776. 11821461 10.1200/JCO.2002.20.3.776

[9] N. Fuchs , S. S. Bielack , D. Epler , P. Bieling , G. Delling , D. Körholz , N. Graf , U. Heise , H. Jürgens , R. Kotz , M. Salzer‐Kuntschik , P. Weinel , M. Werner , K. Winkler , Ann. Oncol. 1998, 9, 893. 9789613 10.1023/a:1008391103132

[10] L. M. Kelley , M. Schlegel , S. Hecker‐Nolting , M. Kevric , B. Haller , C. Rössig , P. Reichardt , L. Kager , T. Kühne , G. Gosheger , R. Windhager , K. Specht , H. Rechl , P. U. Tunn , D. Baumhoer , T. Wirth , M. Werner , T. von Kalle , M. Nathrath , S. Burdach , S. Bielack , I. von Lüttichau , J. Clin. Oncol. 2020, 38, 823. 31928458 10.1200/JCO.19.00827

[11] F. He , W. Zhang , Y. Shen , P. Yu , Q. Bao , J. Wen , C. Hu , S. Qiu , Int. J. Surg. 2016, 36, 283. 27840310 10.1016/j.ijsu.2016.11.016

[12] P. Picci , L. Sangiorgi , B. T. Rougraff , J. R. Neff , R. Casadei , M. Campanacci , J. Clin. Oncol. 1994, 12, 2699. 7989947 10.1200/JCO.1994.12.12.2699

[13] Y. Huang , X. Zhai , T. Ma , M. Zhang , H. Yang , S. Zhang , J. Wang , W. Liu , X. Jin , W. W. Lu , X. Zhao , W. Hou , T. Sun , J. Shen , H. Pan , Y. Du , C. H. Yan , Adv. Mater. 2023, 35, 2300313. 10.1002/adma.202300313 36939167

[14] J. Long , W. Zhang , Y. Chen , B. Teng , B. Liu , H. Li , Z. Yao , D. Wang , L. Li , X. F. Yu , L. Qin , Y. Lai , Biomaterials 2021, 275, 120950. 34119886 10.1016/j.biomaterials.2021.120950

[15] C. Xiao , L. Fan , S. Zhou , X. Kang , P. Guan , R. Fu , C. Li , J. Ren , Z. Wang , P. Yu , Y. Wang , C. Deng , L. Zhou , C. Ning , ACS Nano 2022, 16, 20770. 36412574 10.1021/acsnano.2c07900

[16] D. Zhang , S. Cheng , J. Tan , J. Xie , Y. Zhang , S. Chen , H. Du , S. Qian , Y. Qiao , F. Peng , X. Liu , Bioact. Mater. 2022, 17, 394. 35386440 10.1016/j.bioactmat.2022.01.032 PMC8965036

[17] L. Wang , Q. Yang , M. Huo , D. Lu , Y. Gao , Y. Chen , H. Xu , Adv. Mater. 2021, 33, 2100150. 10.1002/adma.202100150 34146359

[18] B. Yang , J. Yin , Y. Chen , S. Pan , H. Yao , Y. Gao , J. Shi , Adv. Mater. 2018, 30, 1705611. 10.1002/adma.201705611 29333689

[19] C. He , C. Dong , L. Yu , Y. Chen , Y. Hao , Adv. Sci. 2021, 8, e2101739. 10.1002/advs.202101739 PMC8498872 34338444

[20] S. Pan , J. Yin , L. Yu , C. Zhang , Y. Zhu , Y. Gao , Y. Chen , Adv. Sci. 2020, 7, 1901511. 10.1002/advs.201901511 PMC6974945 31993282

[21] C. Yang , H. Ma , Z. Wang , M. R. Younis , C. Liu , C. Wu , Y. Luo , P. Huang , Adv. Sci. 2021, 8, e2100894. 10.1002/advs.202100894 PMC8529444 34396718

[22] J. Zhou , Z. Zhang , J. Joseph , X. Zhang , B. E. Ferdows , D. N. Patel , W. Chen , G. Banfi , R. Molinaro , D. Cosco , N. Kong , N. Joshi , O. C. Farokhzad , C. Corbo , W. Tao , Exploration 2021, 1, 20210011. 37323213 10.1002/EXP.20210011 PMC10190996

[23] Y. Fang , Y. Yu , X. Jiang , P. Liu , Y. Chen , W. Feng , Adv. Funct. Mater. 2023, 33, 2304163.

[24] Y. Zhou , S. Fan , L. Feng , X. Huang , X. Chen , Adv. Mater. 2021, 33, 2104223. 10.1002/adma.202104223 34580933

[25] Z. Tang , Y. Liu , M. He , W. Bu , Angew Chem., Int. Ed. 2019, 58, 946. 10.1002/anie.201805664 30048028

[26] Z. Tang , P. Zhao , H. Wang , Y. Liu , W. Bu , Chem. Rev. 2021, 121, 1981. 33492935 10.1021/acs.chemrev.0c00977

[27] K. Wang , W. Mao , X. Song , M. Chen , W. Feng , B. Peng , Y. Chen , Chem. Soc. Rev. 2023, 52, 6957. 37743750 10.1039/d2cs00435f

[28] L. Pang , R. Zhao , J. Chen , J. Ding , X. Chen , W. Chai , X. Cui , X. Li , D. Wang , H. Pan , Bioact. Mater. 2022, 12, 1. 35087959 10.1016/j.bioactmat.2021.10.030 PMC8777258

[29] M. P. Rayman , Lancet 2012, 379, 1256. 22381456 10.1016/S0140-6736(11)61452-9

[30] J. Köhrle , F. Jakob , B. Contempré , J. E. Dumont , Endocr. Rev. 2005, 26, 944. 16174820 10.1210/er.2001-0034

[31] H. Wang , H. Chen , M. Chernick , D. Li , G. G. Ying , J. Yang , N. Zheng , L. Xie , D. E. Hinton , W. Dong , J. Hazard Mater. 2020, 387, 121720. 31812480 10.1016/j.jhazmat.2019.121720

[32] H. Kim , K. Lee , J. M. Kim , M. Y. Kim , J. R. Kim , H. W. Lee , Y. W. Chung , H. I. Shin , T. Kim , E. S. Park , J. Rho , S. H. Lee , N. Kim , S. Y. Lee , Y. Choi , D. Jeong , Nat. Commun. 2021, 12, 2258. 33859201 10.1038/s41467-021-22565-7 PMC8050258

[33] X. W. Shi , X. Guo , F. L. Ren , J. Li , X. M. Wu , J. Bone Joint Surg. Am. 2010, 92, 72. 20048098 10.2106/JBJS.H.00502

[34] R. Moreno‐Reyes , C. Suetens , F. Mathieu , F. Begaux , D. Zhu , M. T. Rivera , M. Boelaert , J. Nève , N. Perlmutter , J. Vanderpas , N. Engl. J. Med. 1998, 339, 1112. 9770558 10.1056/NEJM199810153391604

[35] R. D. Utiger , N. Engl. J. Med. 1998, 339, 1156. 9770565 10.1056/NEJM199810153391611

[36] K. Bai , B. Hong , J. He , Z. Hong , R. Tan , Int. J. Nanomed. 2017, 12, 4527. 10.2147/IJN.S129958 PMC5485894 28684913

[37] K. Bai , B. Hong , W. Huang , J. He , Pharmaceutics 2020, 12, 774. 31947874 10.3390/pharmaceutics12010043 PMC7022253

[38] G. Deng , K. Niu , F. Zhou , B. Li , Y. Kang , X. Liu , J. Hu , B. Li , Q. Wang , C. Yi , Q. Wang , Sci. Rep. 2017, 7, 43914. 28256626 10.1038/srep43914 PMC5335566

[39] H. Wang , T. Qin , W. Wang , X. Zhou , F. Lin , G. Liang , Z. Yang , Z. Chi , B. Z. Tang , Adv. Sci. 2023, 10, e2301902. 10.1002/advs.202301902 PMC10460872 37357144

[40] J. Xiao , G. Zhang , R. Xu , H. Chen , H. Wang , G. Tian , B. Wang , C. Yang , G. Bai , Z. Zhang , H. Yang , K. Zhong , D. Zou , Z. Wu , Biomaterials 2019, 216, 119254. 31195303 10.1016/j.biomaterials.2019.119254

[41] J. J. O. Garza‐García , J. A. Hernández‐Díaz , J. M. León‐Morales , G. Velázquez‐Juárez , A. Zamudio‐Ojeda , J. Arratia‐Quijada , O. K. Reyes‐Maldonado , J. C. López‐Velázquez , S. García‐Morales , J. Nanobiotechnol. 2023, 21, 252. 10.1186/s12951-023-02027-6 PMC10399041 37537575

[42] L. Mao , L. Wang , M. Zhang , M. W. Ullah , L. Liu , W. Zhao , Y. Li , A. A. Q. Ahmed , H. Cheng , Z. Shi , G. Yang , Adv. Healthcare Mater. 2021, 10, e2100402. 10.1002/adhm.202100402 34050616

[43] M. A. Ruiz‐Fresneda , S. Schaefer , R. Hübner , K. Fahmy , M. L. Merroun , ACS Appl. Mater. Interfaces 2023, 15, 29958. 37294110 10.1021/acsami.3c05100 PMC10316328

[44] J. Han , X. Guo , Y. Lei , B. S. Dennis , S. Wu , C. Wu , Carbohydr. Polym. 2012, 90, 122. 24751019 10.1016/j.carbpol.2012.04.068

[45] A. Kumar , I. Sevonkaev , D. V. Goia , J. Colloid Interface Sci. 2014, 416, 119. 24370410 10.1016/j.jcis.2013.10.046

[46] K. Li , Q. Xu , S. Gao , S. Zhang , Y. Ma , G. Zhao , Y. Guo , J. Hazard Mater. 2021, 414, 125545. 33667801 10.1016/j.jhazmat.2021.125545

[47] T. Hu , Z. Gu , G. R. Williams , M. Strimaite , J. Zha , Z. Zhou , X. Zhang , C. Tan , R. Liang , Chem. Soc. Rev. 2022, 51, 6126. 35792076 10.1039/d2cs00236a

[48] T. Hu , X. Mei , Y. Wang , X. Weng , R. Liang , M. Wei , Sci. Bull. 2019, 64, 1707. 10.1016/j.scib.2019.09.021 36659785

[49] W. Shen , T. Hu , X. Liu , J. Zha , F. Meng , Z. Wu , Z. Cui , Y. Yang , H. Li , Q. Zhang , L. Gu , R. Liang , C. Tan , Nat. Commun. 2022, 13, 3384. 35697679 10.1038/s41467-022-31106-9 PMC9192653

[50] T. Hu , W. Shen , F. Meng , S. Yang , S. Yu , H. Li , Q. Zhang , L. Gu , C. Tan , R. Liang , Adv. Mater. 2023, 35, 2209692. 10.1002/adma.202209692 36780890

[51] Y. Yang , T. Hu , Y. Bian , F. Meng , S. Yu , H. Li , Q. Zhang , L. Gu , X. Weng , C. Tan , R. Liang , Adv. Mater. 2023, 35, 2211205. 10.1002/adma.202211205 36913539

[52] L. Peng , X. Mei , J. He , J. Xu , W. Zhang , R. Liang , M. Wei , D. G. Evans , X. Duan , Adv. Mater. 2018, 30, 1707389. 10.1002/adma.201707389 29537662

[53] X. Mei , T. Hu , H. Wang , R. Liang , W. Bu , M. Wei , Biomaterials 2020, 258, 120257. 32798739 10.1016/j.biomaterials.2020.120257

[54] T. Hu , L. Yan , Z. Wang , W. Shen , R. Liang , D. Yan , M. Wei , Chem. Sci. 2021, 12, 2594. 34164027 10.1039/d0sc06742c PMC8179329

[55] Z. Lv , T. Hu , Y. Bian , G. Wang , Z. Wu , H. Li , X. Liu , S. Yang , C. Tan , R. Liang , X. Weng , Adv. Mater. 2023, 35, 2206545. 10.1002/adma.202206545 36426823

[56] G. Wang , Z. Lv , T. Wang , T. Hu , Y. Bian , Y. Yang , R. Liang , C. Tan , X. Weng , Adv. Sci. 2022, 10, e2204234. 10.1002/advs.202204234 PMC9811441 36394157

[57] Y. Wang , S. Shen , T. Hu , G. R. Williams , Y. Bian , B. Feng , R. Liang , X. Weng , ACS Nano 2021, 15, 9732. 34086438 10.1021/acsnano.1c00461

[58] Y. Bian , X. Cai , Z. Lv , Y. Xu , H. Wang , C. Tan , R. Liang , X. Weng , Adv. Sci. 2023, 10, e2301806. 10.1002/advs.202301806 PMC10460877 37329200

[59] Z. Zhou , T. Wang , T. Hu , H. Xu , L. Cui , B. Xue , X. Zhao , X. Pan , S. Yu , H. Li , Y. Qin , J. Zhang , L. Ma , R. Liang , C. Tan , Adv. Mater. 2024, 36, 2311002. 10.1002/adma.202311002 38408758

[60] B. Li , D. Chu , H. Cui , Z. Li , Z. Zhou , C. Tan , J. Li , SmartMat 2023, 4, e1243.

[61] X. Zhao , H. Qiu , Y. Shao , P. Wang , S. Yu , H. Li , Y. Zhou , Z. Zhou , L.‐F. Ma , C. Tan , Acta Phys‐Chim. Sin. 2023, 39, 2211043.

[62] L. Zong , Y. Yu , J. Wang , P. Liu , W. Feng , X. Dai , L. Chen , C. Gunawan , S. L. J. Yun , R. Amal , S. Cheong , Z. Gu , Y. Chen , Biomaterials 2023, 296, 122074. 36889145 10.1016/j.biomaterials.2023.122074

[63] F. Melin , P. Hellwig , Chem. Rev. 2020, 120, 10244. 32820893 10.1021/acs.chemrev.0c00249

[64] Y. Wang , R. Branicky , A. Noë , S. Hekimi , J. Cell Biol. 2018, 217, 1915. 29669742 10.1083/jcb.201708007 PMC5987716

[65] F. Chen , H. Di , Y. Wang , Q. Cao , B. Xu , X. Zhang , N. Yang , G. Liu , C. G. Yang , Y. Xu , H. Jiang , F. Lian , N. Zhang , J. Li , L. Lan , Nat. Chem. Biol. 2016, 12, 174. 26780405 10.1038/nchembio.2003

[66] I. Domonkos , M. Kis , Z. Gombos , B. Ughy , Prog. Lipid Res. 2013, 52, 539. 23896007 10.1016/j.plipres.2013.07.001

[67] A. Saraswathibhatla , D. Indana , O. Chaudhuri , Nat. Rev. Mol. Cell. Biol. 2023, 24, 495. 36849594 10.1038/s41580-023-00583-1 PMC10656994

[68] H. Kang , A. L. Strong , Y. Sun , L. Guo , C. Juan , A. C. Bancroft , J. H. Choi , C. A. Pagani , A. A. Fernandes , M. Woodard , J. Lee , S. Ramesh , A. W. James , D. Hudson , K. N. Dalby , L. Xu , R. J. Tower , B. Levi , Bone. Res. 2024, 12, 17. 38472175 10.1038/s41413-024-00320-0 PMC10933265

[69] B. Amulic , C. Cazalet , G. L. Hayes , K. D. Metzler , A. Zychlinsky , Annu. Rev. Immunol. 2012, 30, 459. 22224774 10.1146/annurev-immunol-020711-074942

