# Supplementary material for: A Se Nanoparticle/MgFe‐LDH Composite Nanosheet as a Multifunctional Platform for Osteosarcoma Eradication, Antibacterial and Bone Reconstruction
Source: Adv Sci (Weinh). 2024 Jul 3;11(33):2403791. doi: 10.1002/advs.202403791 (PMC11434235; doi:10.1002/advs.202403791)
Supplement: Supplementary file 1 — Supporting Information [file ADVS-11-2403791-s001.docx]

***Supporting Information***

**A Se nanoparticle/MgFe-LDH composite nanosheet as a multifunctional platform for osteosarcoma eradication, antibacterial and bone reconstruction**

*Yixin Bian, Kexin Zhao, Tingting Hu, Chaoliang Tan,* Ruizheng Liang,* and Xisheng Weng**

These authors contributed equally: Yixin Bian, Kexin Zhao, and Tingting Hu

Y. Bian and Prof. X. Weng

Department of Orthopedic Surgery, State Key Laboratory of Complex Severe and Rare Diseases, Peking Union Medical College Hospital, Chinese Academy of Medical Science and Peking Union Medical College, Beijing 100730, China

E-mail: [xshweng@pumc.cams.cn](mailto:xshweng@pumc.cams.cn) (X. Weng)

K. Zhao and Prof. R. Liang

State Key Laboratory of Chemical Resource Engineering, Beijing Advanced Innovation Center for Soft Matter Science and Engineering, Beijing University of Chemical Technology, Beijing 100029, P. R. China.

E-mail: [liangrz@mail.buct.edu.cn](mailto:liangrz@mail.buct.edu.cn) (R. Liang)

T. Hu and Prof. C. Tan

Department Electrical and Electronic Engineering, The University of Hong Kong, Pokfulam Road, Hong Kong SAR 999077, P. R. China.

E-mail: [cltan@hku.hk](mailto:cltan@hku.hk) (C. Tan)

Prof. R. Liang

Quzhou Institute for Innovation in Resource Chemical Engineering, Quzhou 324000, P. R. China.

**Experimental Section**

***Materials:*** Ferric nitrate nonahydrate (Fe(NO_3_)_3_·9H_2_O, 99.0%), magnesium nitrate hexahydrate (Mg(NO_3_)_2_·6H_2_O, 99.0%), sodium nitrate (NaNO_3_), and sodium hydroxide (NaOH) were purchased from Aladdin Industrial Corporation (Shanghai, China). Na_2_SeO_3_ was bought from Sigma-Aldrich Corporation (Shanghai, China). GSH and L-cysteine (L-Cys) were obtained from Macklin Biochemical Co., Ltd (Shanghai, China). TMB, TA, and DMPO were gained from J&K Scientific (Beijing, China).

***Characterizations:*** Shimadzu XRD-6000 diffractometer (Cu Kα source) was used to record XRD patterns of samples with the scan range from 3° to 70° and the scan step of 0.02°. The morphology of samples was characterized using TEM (JEOL, JEM-2100, 200 kV). Shimadzu U-3000 spectrophotometer was employed to record UV absorption spectra of TMB (slit width: 1.0 nm). ESR spectra were acquired on the Bruker EMX1598 spectrometer. The thickness of the nanosheets was characterized by AFM (MultiMode 8, Bruker). The element valence state of the samples was investigated by XPS (Escalab 250Xi, Thermo Scientific, USA). The contents of elements were detected by ICP-AES (Shimadzu ICPS-7500).

***Synthesis of MgFe-LDH nanosheets:*** MgFe-LDH nanosheets were synthesized using a co-precipitation method. Briefly, solution A: Mg(NO_3_)_2_·6H_2_O (0.2564 g) and Fe(NO_3_)_3_·9H_2_O (0.202 g) were dissolved in deionized water (50 mL). Solution B: NaNO_3_ (0.0425 g) was dissolved in deionized water (50 mL). Solution C: NaOH (0.3 g) was dissolved in deionized water (50 mL). Solution A and C were slowly added to solution B and stirred at room temperature for 30 min. The resulting MgFe-LDH nanosheets were centrifuged and washed three times at 7000 rpm for 3 min.

***Synthesis of LDH/Se nanosheets:*** LDH/Se nanosheets were prepared through in situ reduction method. In brief, solution A: Na_2_SeO_3_ (0.0692 g) was dissolved in deionized water (4 mL). Solution B: L-Cys (0.0969 g) was dissolved in deionized water (4 mL). Subsequently, 0.5 mL solution A was slowly added to 10 mL MgFe-LDH nanosheet suspension (2 mg mL^−1^) and stirred at room temperature for 8 h. Then, 1 mL solution B was slowly added to the above suspension and stirred at room temperature for 30 min. The resulting LDH/Se nanosheets were centrifuged and washed three times at 7000 rpm for 3 min.

***Synthesis of BGS@LDH/Se composite scaffolds:*** BGS were placed in LDH/Se suspension (1 mg mL^−1^) with ultrasound treatment for 5 min, and then thoroughly dried in a 60 ℃ oven. The whole process was repeated three times.

***GSH*** ***depletion:*** The GSH consumption ability of LDH/Se was evaluated by DTNB assay. DTNB solution (2 mg mL^−1^, 200 μL), LDH/Se suspension (1 mg mL^−1^, 0, 40, 80, 200 and 400 μL) and GSH solution (10 mM, 200 μL) were added to deionized water (3600, 3560, 3520, 3400 and 3200 μL, respectively). After reaction for 1 h, the mixture was centrifuged and the supernatant was measured using a UV-vis spectrophotometer to record the absorbance at 412 nm.

***ROS detection:*** TMB and TA were used as ·OH probes to evaluate the CDT performance of LDH/Se. For TMB assay, LDH/Se (100 μg mL^−1^), TMB (0.8 mM) and H_2_O_2_ (1 mM) were added to PBS at pH=7.4 and 6.5, respectively. The absorbance of mixture at λ = 652 nm was monitored every two minutes to indicate the ·OH generation. For TA assay, LDH/Se (200 μL, 1 mg mL^−1^) and H_2_O_2_ (20 μL, 100 mM) were mixed with TA solution (200 μL, 30 mM). The ·OH generation was indirectly detected by measuring the fluorescence intensity of mixture at 420 nm every two minutes.

The ·OH-generating activity of LDH/Se was further verified by ESR spectroscopy using DMPO as a probe. In brief, 20 μL DMPO, 20 μL LDH/Se (500 μg mL^−1^), and 20 μL H_2_O_2_ (5 mM) were added to 40 μL PBS at pH=7.4 and 6.5, respectively. Then, the mixture was exposed to ESR to capture characteristic 1: 2: 2: 1 ·OH signal.

To determine the Michaelis–Menten kinetics of LDH/Se, LDH/Se (100 μg mL^−1^), TMB (0.8 mM), and different concentrations of H_2_O_2_ (0.2, 0.5, 1, 1.5, and 2 mM) were added to PBS at pH=6.5, respectively. The Michaelis–Menten kinetic curve was acquired by plotting the initial velocity against H_2_O_2_ concentration, the *V*_max_ and *K*_M_ were calculated by Lineweaver–Burk plot.

***Metal ions release:*** BGS@LDH/Se was dispersed into 8 mL PBS and incubated at 37 ℃ for 35 days. An aliquot (1 mL) of dispersion was collected at each specified time point and analyzed by ICP-AES.

***In situ osteosarcoma recurrence prevention:*** Luciferase labeled-human osteosarcoma cells (143B/LUC, 1.5 × 10^6^/50 μL) were subperiosteally injected into the tibia of BALB/c nude mice along with 50 μL matrix glue to build an *in situ* osteosarcoma model. Two weeks after model establishment, the osteosarcomas were surgically excised with no extensive resection of the tumor margin was performed. The bone defects resulting from osteosarcoma resection were implanted with BGS, BGS@LDH, or BGS@LDH/Se (designed as 1.5 mm × 1.5 mm × 1 mm). The negative control group (NC) group did not receive initial subperiosteal osteosarcoma cell injection (replaced as pristine matrix glue injection) but underwent tibial bone defect modeling with no scaffold implantation. The positive control group (PC) group underwent the same initial subperiosteal osteosarcoma cell injection followed by osteosarcoma resection but without scaffold implantation. *In vivo* imaging of small animals was conducted 4, 8, 12, and 16 after scaffold implantation with Caliper IVIS Lumina II. Tumor volume and body weight of mice in different groups were recorded every 2 days. The survival rate of mice was monitored up to 40 days after scaffold implantation. Another paralleled experiment with the same protocols was conducted simultaneously, of which the osteosarcoma-bearing limbs of mice were harvested at day 16 and imaged with a digital camera. Afterward, the osteosarcoma and surrounding tissues were fixed in paraformaldehyde and sliced for H&E and Ki-67 staining.

***In vitro antibacterial assays:*** *MRSA* at a concentration of 10^7^ CFU/mL was co-incubated with a 1 mL suspension of LDH or LDH/Se at various concentrations (0, 25, 50, 100, and 200 μg mL^−1^) for 12 h. Subsequently, the bacteria were diluted and inoculated onto LB agar plates, which were then subjected to a humidified incubator at a constant temperature of 37°C for 12 h. The resulting bacterial colonies were imaged using a digital camera (Fujifilm, Tokyo, Japan) and counted with ImageJ 1.52v software. To evaluate the antibacterial efficiency of LDH/Se more directly, 10^8^ CFU/mL bacteria were dropped onto BGS, BGS@LDH, or BGS@LDH/Se and allowed for 12 h adhesion and treatment. The morphology of bacteria in different groups was recorded under a SEM (Zeiss, Jena, Germany) after being dehydrated in a gradient of ethanol (30%, 50%, 70%, 90%, and 100%).

***In vivo periprosthetic infection treatment:*** A rabbit tibia defect model was established by surgically inducing a full-thickness circular defect with a diameter of 6 mm in the upper tibia of New Zealand rabbits, where the experimental procedures strictly adhered to the National Institutes of Health Guidelines for the use of experimental animals, and all protocols were approved by the Animal Care and Use Committee of Peking Union Medical College Hospital with the ethics number of XHDW-2023-021. To simulate clinical postoperative periprosthetic infection, BGS, BGS@LDH, and BGS@LDH/Se were immersed in a *MRSA* suspension (10^7^ CFU/mL) for 2 h prior to implantation into the tibia defects. One month after implantation, the tibias of the New Zealand white rabbits were harvested and fixed in paraformaldehyde for Micro-CT imaging and 3D reconstruction (Siemens system, Erlangen, Germany). In addition, muscle tissues (100 mg per rabbit) surrounding the scaffolds were collected, homogenized, and cultured on an agar medium to observe bacterial colony formation. The resulting colonies were documented using a digital camera (Fujifilm, Tokyo, Japan) and quantified with ImageJ 1.52v software.

***RNA sequence for MRSA:*** *MRSA* cultures were exposed to regular medium or medium containing 100 µg mL^−1^ LDH/Se for a duration of 12 h, after which the bacterial samples were collected for total RNA extraction using TRIzol reagent (Invitrogen, CA, USA). RNA sequencing was performed based on the TruSeq PE Cluster Kit v3-cBot-HS (Illumina, CA, USA), following the manufacturer's instructions. Differential expression analysis was conducted utilizing the DESeq R package (version 1.18.0) with |log2FC | > 1 (p-value < 0.05) as the criteria of differentially expressed genes. GO and KEGG analysis were involved in differentially expressed gene enrichment.

***In vitro Biocompatibility evaluation:*** To assess the biocompatibility of BGS, BGS@LDH, and BGS@LDH/Se, the 24-well Transwell plates (3 µm diameter pores, Corning, NY, USA) were involved, where the upper chamber was placed with BGS, BGS@LDH, or BGS@LDH/Se and a control group was included without no scaffold in upper chamber. In the lower chamber, ten thousand hBMSCs were seeded and the cell culture medium was replaced daily. The cellular proliferation activities were assessed on specific days (1, 3, 5, and 7) with a Live/Dead staining kit (Sigma, St Louis, MO, USA) and a CCK-8 kit (Sigma, St Louis, MO, USA). The Live/Dead staining was observed under a fluorescence microscopy (Olympus, Tokyo, Japan). For the CCK-8 assays, the optical density (OD) values at 450 ± 5 nm were measured using a multifunctional full-wavelength microplate reader (Varioskan Flash, Thermo Fisher Scientific, USA). Moreover, fresh rabbit blood (100 μL) was incubated with the leachates of BGS, BGS@LDH, and BGS@LDH/Se (1mL). Water was used as the positive control and PBS as the negative control, respectively. After incubation at 37 °C for 4 h, centrifuge the supernatant to determine the absorbance at 570 nm. To assess the biocompatibility of the scaffold in a more intuitive manner, a total of ten thousand hBMSCs were directly seeded onto the surfaces of BGS, BGS@LDH, and BGS@LDH/Se in 96-well plates and allowing the cells to adhere for 24 h. Then the cell-seeded scaffolds were fixed with paraformaldehyde and glutaraldehyde followed by staining with DAPI and rhodamine-phalloidin (Sigma, St Louis, MO, USA). The stained cell-seeded scaffolds were further transferred to a culture dish designed specifically for confocal laser scanning microscopy (CLSM) and scanned under a CLSM (Leica, Wetzlar, Germany) to obtain detailed images of cellular morphology and attachment.

***In vitro osteogenic properties evaluation******:*** To evaluate the osteogenic potential of BGS, BGS@LDH, and BGS@LDH/Se, an initial population of 20,000 hBMSCs was cultured on the lower chamber of a 24-well Transwell plate with 3 µm diameter pores (Corning, NY, USA) for 24 h for cell adhesion and proliferation. Subsequently, the BGS, BGS@LDH, or BGS@LDH/Se were introduced into the upper chamber of the Transwell plate, serving as inducers of osteogenic differentiation for hBMSCs. By contrast, the control group lacked any scaffold in the upper chamber and only underwent daily medium replacement. Fourteen days after co-culture, the hBMSCs were fixed with paraformaldehyde and subjected to ALP or ARS staining for 30 min, which were observed under an optimal inverted microscope (Olympus, Tokyo, Japan) to assess alkaline phosphatase activities and calcium deposition. Quantitative analysis of randomly selected microscopic fields of ALP and ARS staining was further performed using ImageJ 1.52v software.

***In vivo osteogenic assays:*** The osteogenic capabilities of BGS, BGS@LDH, and BGS@LDH/Se were assessed using critical-sized calvaria defect models of New Zealand White Rabbits. All surgical procedures strictly adhered to the National Institutes of Health Guidelines for experimental animal use, following the Guide for the Animal Care and Use Committee of Peking Union Medical College Hospital with the ethics number of XHDW-2023-021. Specifically, four circular defects with a diameter of 6 mm were created in the rabbit skulls and subsequently filled with BGS, BGS@LDH, and BGS@LDH/Se, while the control group had nothing implanted in the defect. To track the regeneration of bone tissue, fluorescent ARS (Sigma, St Louis, MO, USA) was intraperitoneally injected at 4 and 6 weeks post-scaffold implantation. Four and eight weeks after surgery, the rabbits were euthanized and the skulls were harvested for Micro-CT scanning and 3D reconstruction utilizing a Siemens system (Erlangen, Germany) to evaluate bone regeneration. Furthermore, the skulls were decalcified, embedded in paraffin, and sectioned for H&E and Sirius Red staining, as well as collagen I immunofluorescent staining to provide detailed insights into the osteogenic properties of BGS, BGS@LDH, and BGS@LDH/Se. An optimal inverted microscope (Olympus, Tokyo, Japan) and laser scanning confocal microscope (Leica, Wetzlar, Germany) were utilized to observe and image the sections.

***Transcriptome sequencing of hBMSCs:*** One hundred thousand hBMSCs were co-cultured on the lower chamber of a 6-well Transwell plate (3 µm diameter pores) for 7 days with or without the BGS@LDH/Se in the upper chamber. To eliminate DNA contamination, an RNA extraction kit along with DNase (Thermo Fisher, USA) was employed, which was then enriched by magnetic beads with Oligo (dT) and interrupted with specialized reagents to serve as templates for subsequent steps. Utilizing six-base random primers and a two-strand reaction system, both single-strand cDNA and double-strand cDNA were synthesized, allowing for amplification of selected fragment sizes. The Agilent 2100 Bioanalyzer was utilized to provide a comprehensive assessment of the constructed library. Finally, Illumina sequencing technology was employed to generate high-throughput sequencing data. R Foundation for Statistical Computing (version 4.2.2; Vienna, Austria) was employed for data analyses and image production.

***qRT-PCR analysis*:** The RNA extraction method followed the Transcriptome Sequencing section. A NanoDrop spectrophotometer (Thermo Fisher Scientific, USA) measured the RNA concentration, and the RNA was then transcribed into cDNA using an RNA-to-cDNA kit (Applied Biosystems, Foster City, USA). RT-PCR was performed on the ABI Step One Plus real-time PCR system (Applied Biosystems, USA) with an SYBR Green RT-PCR kit (Takara, Japan) for target gene detection. GAPDH served as the reference gene, and the ∆∆cT method determined relative gene expression.

***Western blot assay******:*** BGS, BGS@LDH, or BGS@LDH/Se were placed on the upper chamber of a 6-well Transwell plate with 3 µm diameter pores (Corning, NY, USA) while 100,000 hBMSCs were cultured in the lower chamber. Seven days after co-culture, the cells were digested and washed with PBS three times for sample preparation. Total proteins were extracted using RIPA Lysis and Extraction Buffer (Invitrogen, Carlsbad, CA), and the concentration was determined using the PierceTM Rapid Gold BCA Protein Assay Kit (Invitrogen, Carlsbad, CA). To facilitate protein electrophoresis, the extracted proteins were transferred onto a polyvinylidene difluoride (PVDF) membrane and subsequently blocked with a 5% dilute skim milk solution. After washing the membrane with Tris Buffered Saline Tween (TBST), primary antibodies were applied and incubated overnight at 4 °C, followed by secondary antibody incubation at room temperature for 30 min. Visualization of the target bands was achieved using enhanced chemiluminescence (ECL, Thermo Fisher Scientific, CA, USA), and quantitative analysis was performed using ImageJ 1.52v software.

***Immunofluorescence*:** Proteinase K (Sigma-Aldrich) and 3% H_2_O_2_ were utilized to digest and eliminate endogenous peroxidase on the skull slide. After blocking with donkey serum, the slides were incubated with the primary antibody overnight at 4 °C, and then with the secondary antibody for 60 minutes at room temperature. Fluorescent images were captured using CLSM (Leica, Wetzlar, Germany).

***Single-cell RNA-sequencing analysis:*** Single-cell RNA-sequencing analysis was conducted on New Zealand White Rabbits with two critical-sized calvaria defects that were implanted with BGS and BGS@LDH/Se. The experimental animals were sacrificed two weeks post-scaffold implantation, and the regenerated tissues from the calvarial defect area were harvested. The collected tissues underwent multiple washes in cold PBS and were subsequently enzymatically digested at 37 °C for 40 min. To isolate single cells, a 70 μm nylon mesh was employed to filter the resulting mixture. Subsequently, mRNA libraries were then prepared and sequenced. Cells meeting specific criteria, including over 300 detectable genes, a minimum of 500 read counts, and less than 20% mitochondrial gene expression, were retained. The retained cells underwent log-normalization and scaling using the Seurat pipeline. t-distributed Stochastic Neighbor Embedding (t-SNE) was employed to visualize cellular diversity in reduced dimensions.

***Statistical analysis:*** Data are expressed as mean ± standard deviation (S.D.). Statistical comparisons were made by one-way ANOVA (for multiple comparisons): *p < 0.05, **p < 0.01, *** p < 0.001, **** p < 0.0001.

**
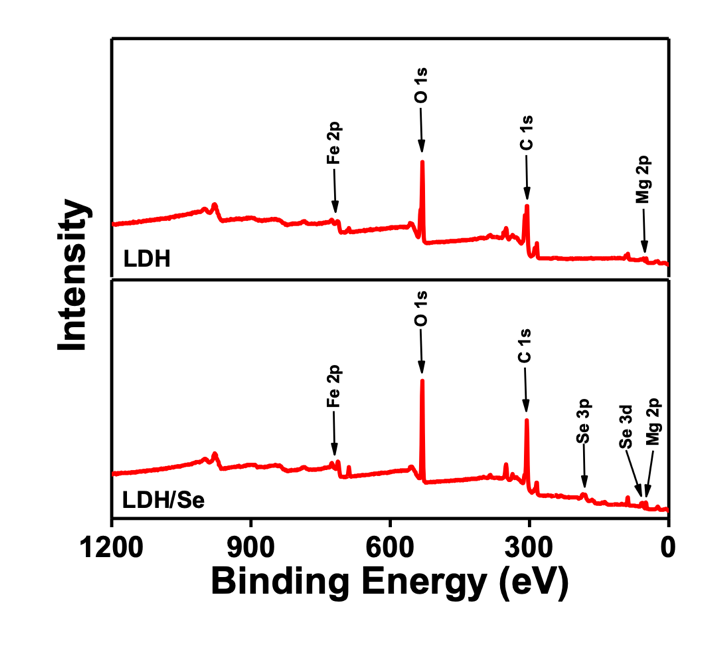
**

**Figure S1.** XPS spectra of MgFe-LDH and LDH/Se nanosheets.

**
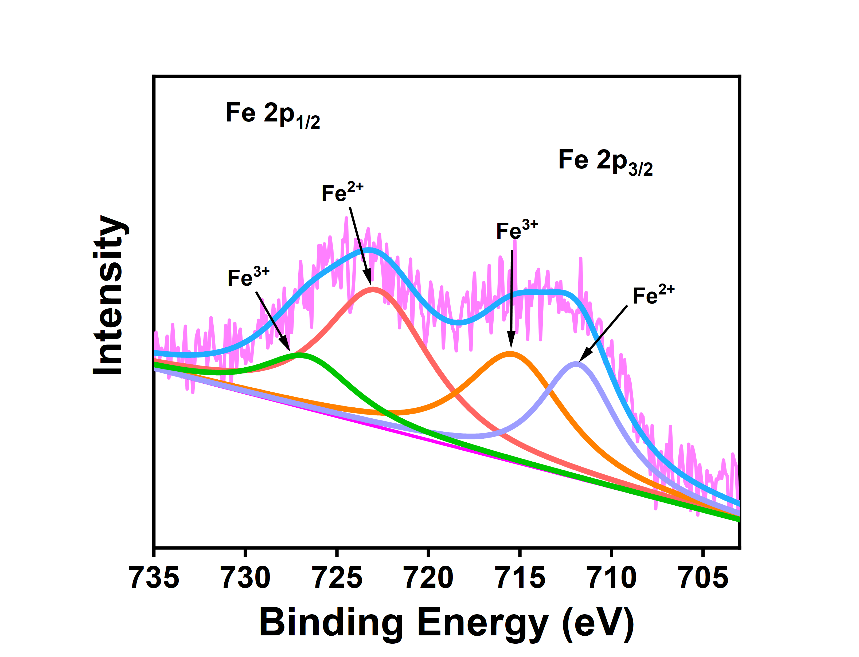
**

**Figure S2.** XPS Fe 2*p* spectra of LDH/Se nanosheets after reaction with GSH.

**
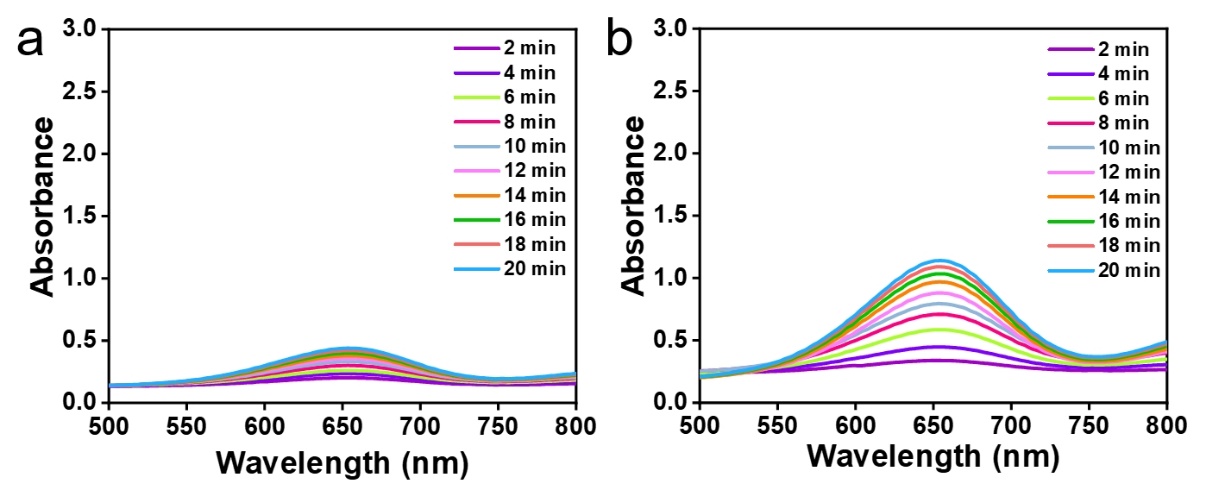
**

**Figure S3.** The UV absorbance spectra of TMB in the presence of LDH/Se and H_2_O_2_ at a) pH 7.4 and b) pH 6.5.

**
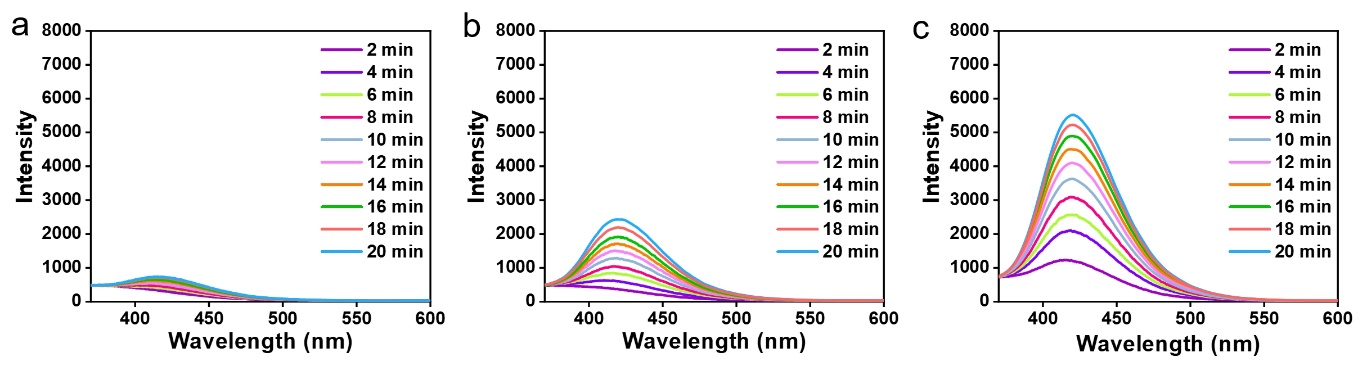
**

**Figure S4.** The fluorescence spectra of TA in the presence of LDH/Se and H_2_O_2_ at a) pH 7.4, b) pH 6.5, and c) pH 6.5 with GSH.

**
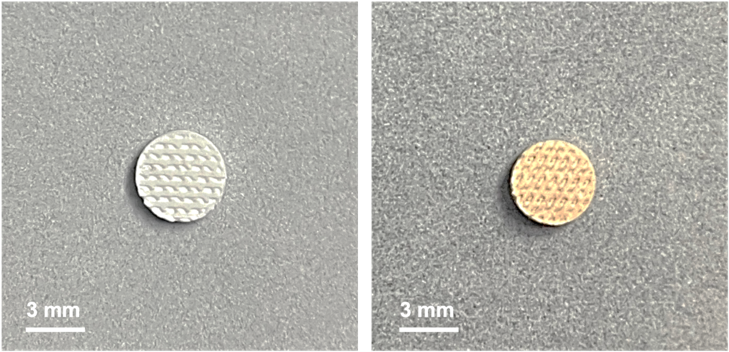
**

**Figure S5.** Digital photos of BGS (left) and BGS@LDH/Se (right) scaffolds.

**
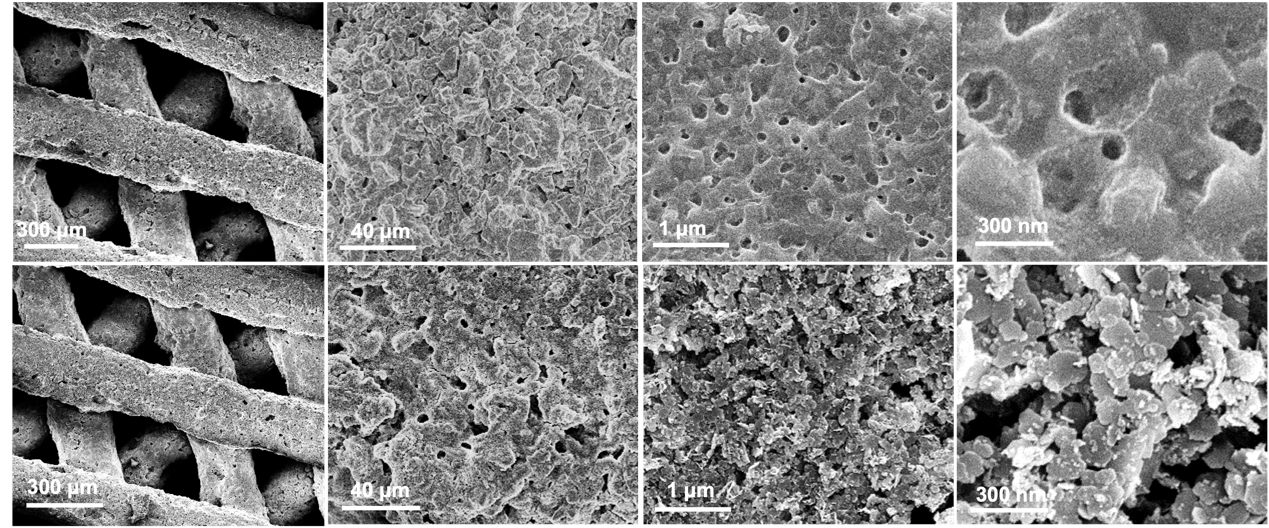
**

**Figure S6.** SEM images of BGS (top) and BGS@LDH/Se scaffolds (below).

**
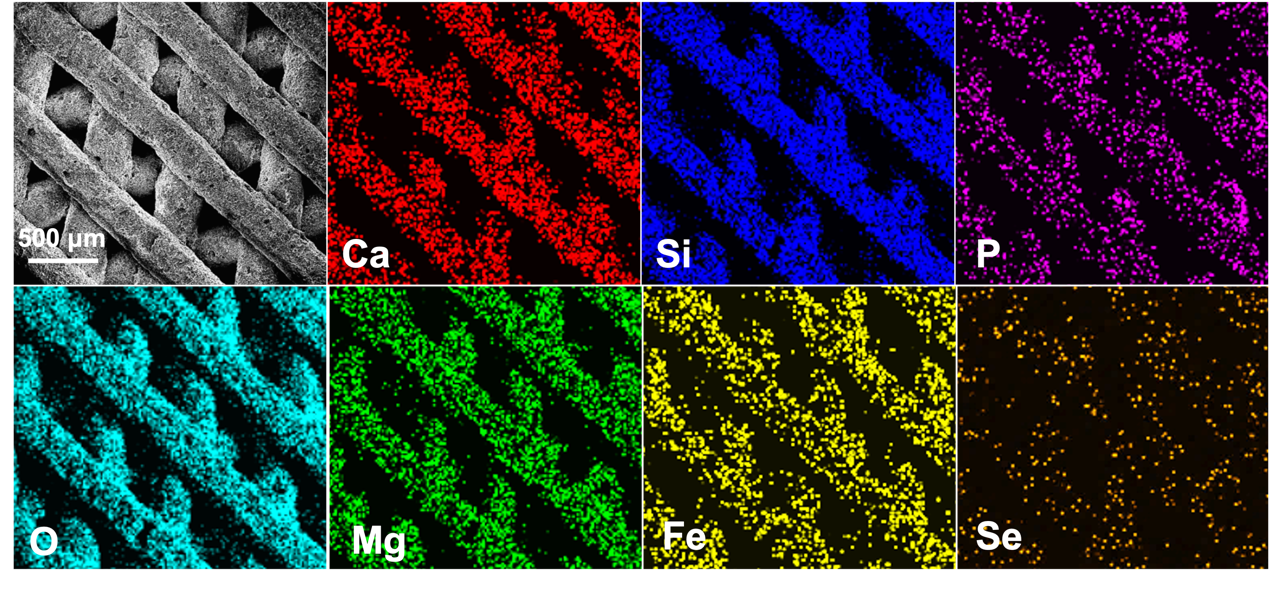
**

**Figure S7.** EDS elemental mapping of BGS@LDH/Se scaffold.


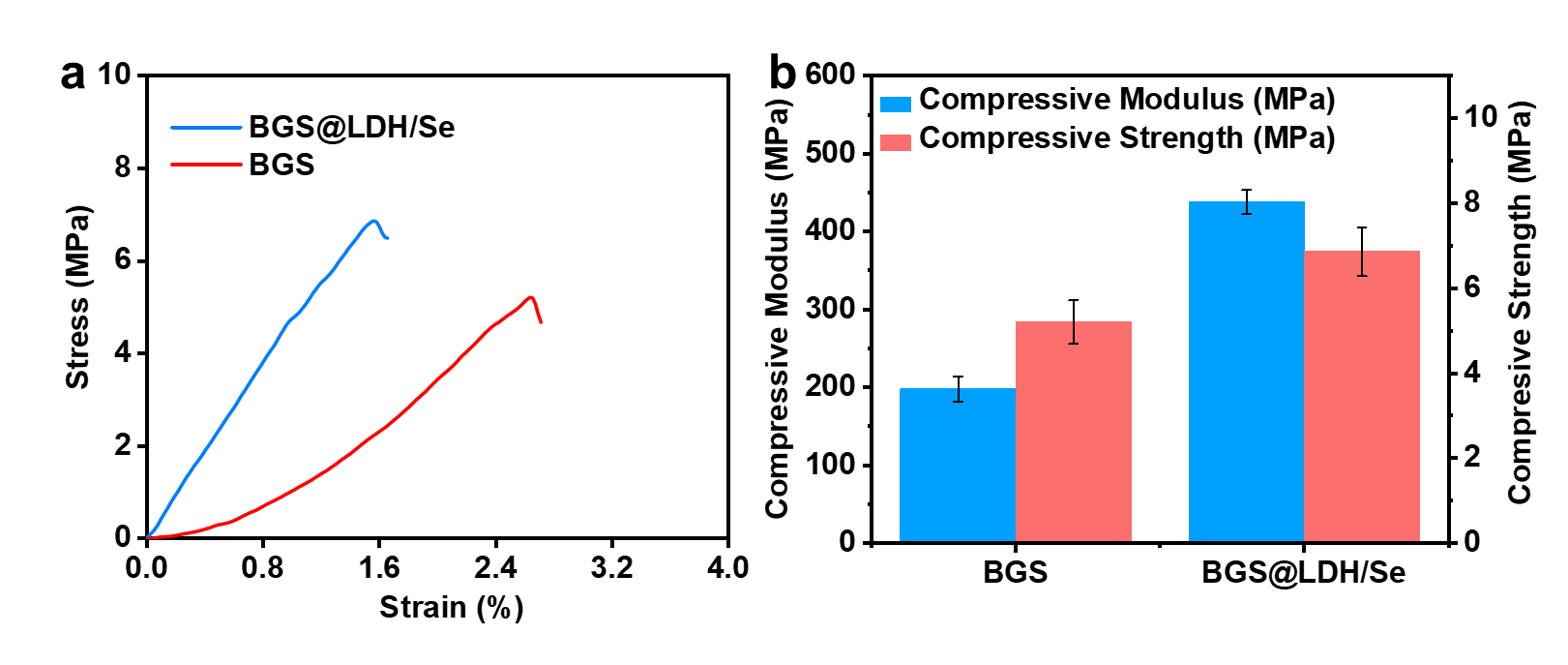


**Figure S8.** a) Stress-strain curve, b) compression modulus and strength of scaffolds before and after LDH/Se deposition.

**
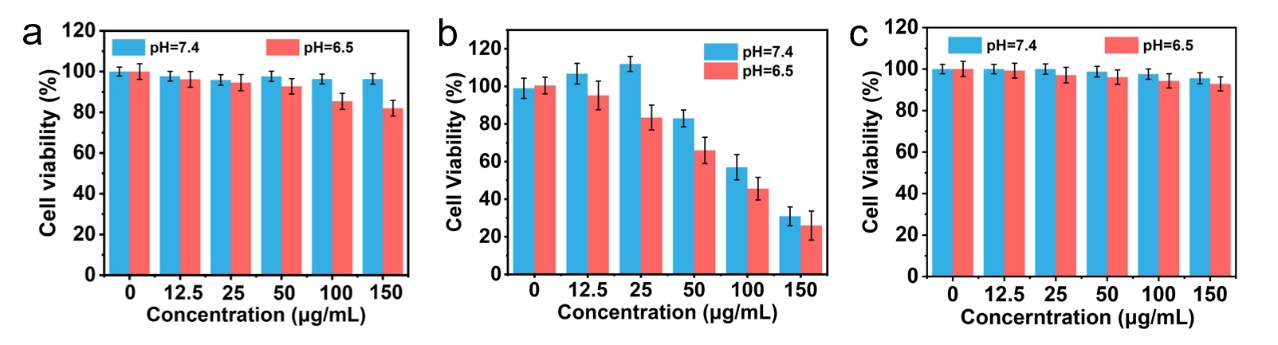
**

**Figure S9.** MTT experiments evaluating the biocompatibility of a) LDH, b) Na_2_SeO_3_, and c) LDH/Se.

**
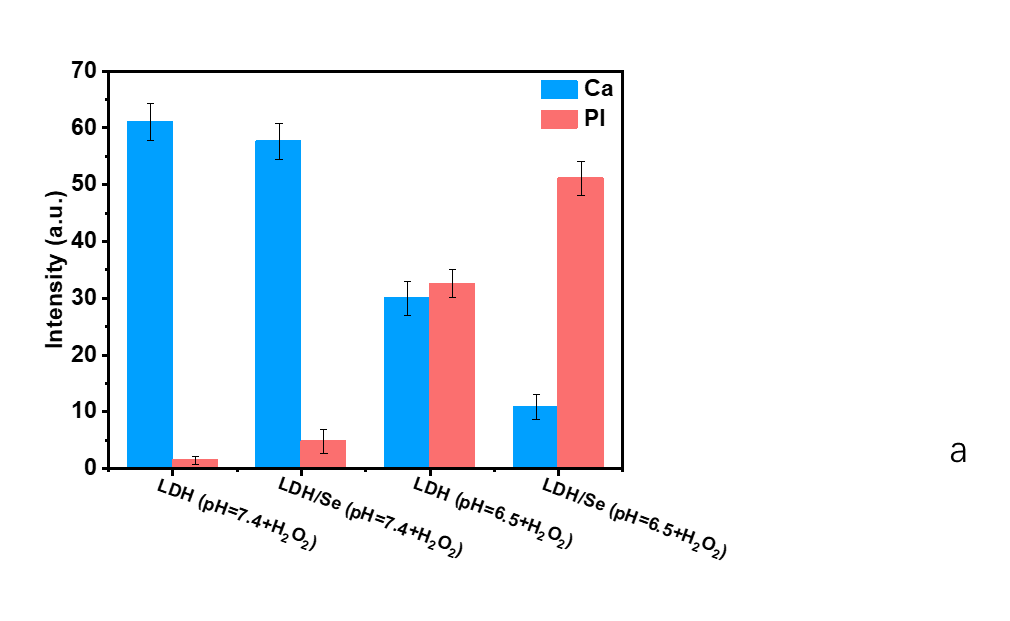
**

**Figure S10.** Quantitative analysis of Calcein-AM/PI double staining images.


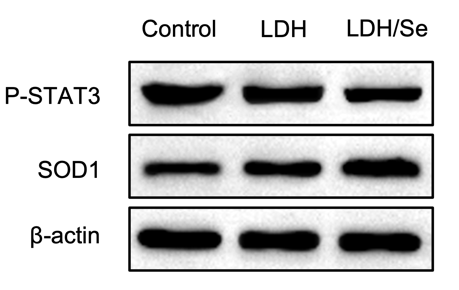


**Figure S11.** Western blot assays detecting SOD-1 and p-STAT3 protein expression of Saos-2 cells treated with indicated strategies.


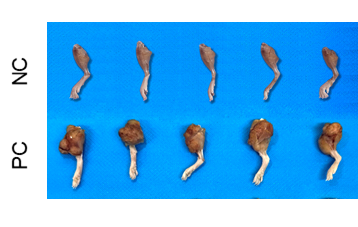


**Figure S12.** Gross image of the knee and surrounding tissues of osteosarcoma-bearing mice in NC (negative control group) and PC (positive control group) groups.


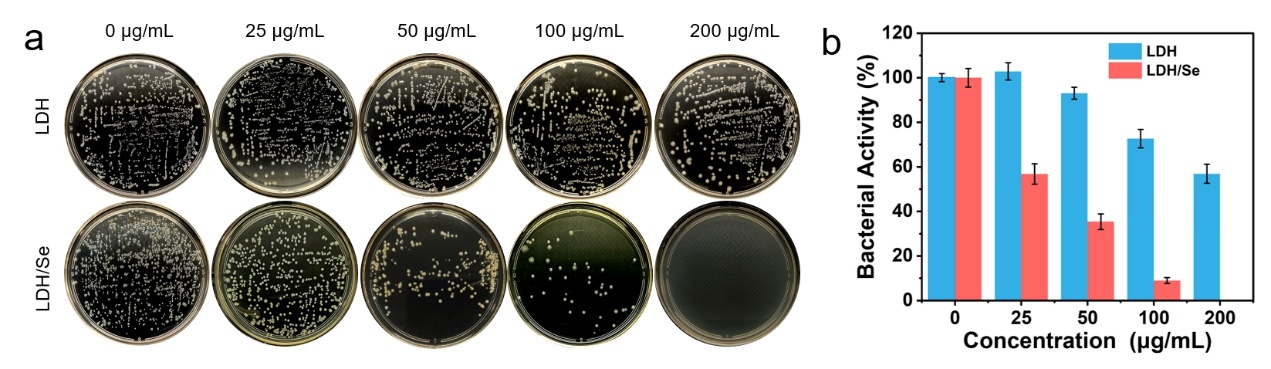


**Figure S13.** a) Qualitative and b) quantitative analysis of the *E. coli* colonies pretreated with LDH or LDH/Se at indicated concentrations.


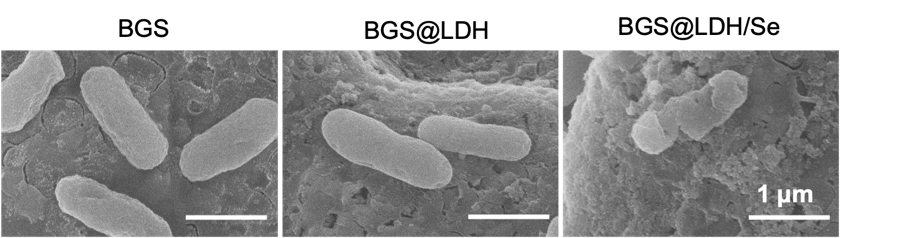


**Figure S14.** SEM images of *E. coli* inoculated on BGS, BGS@LDH, and BGS@LDH/Se.


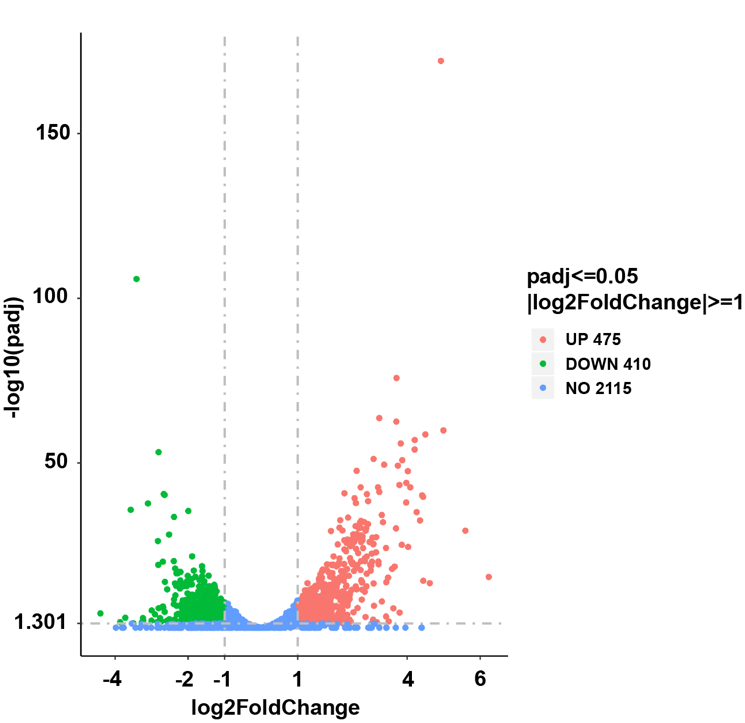


**Figure S15.** Volcano map illustrating the differentially expressed genes of *MRSA* between LDH/Se group and control group. (padj <= 0.05&|log2 Fold Change| > 1).

**
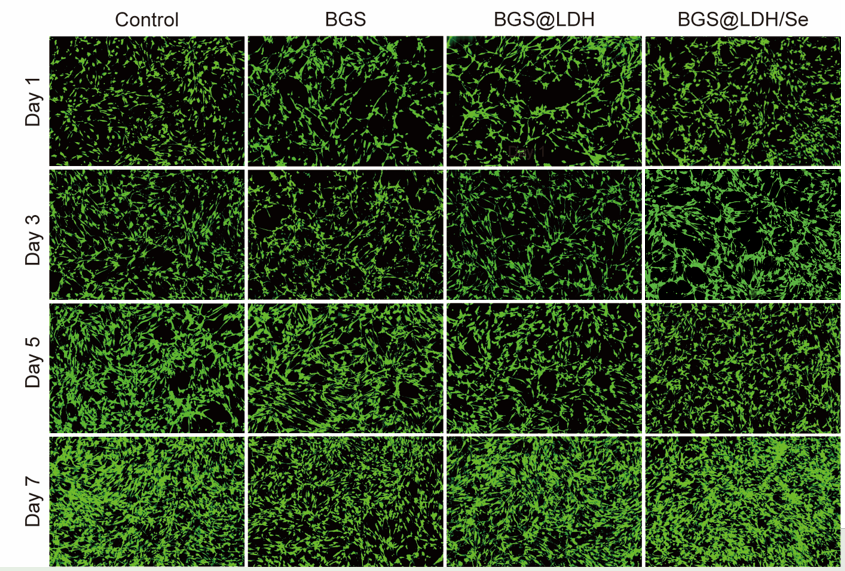
**

**Figure S16.** Live/Dead staining of hBMSCs incubated with regular medium (Control), BGS, BGS@LDH, or BGS@LDH/Se at indicated time points.


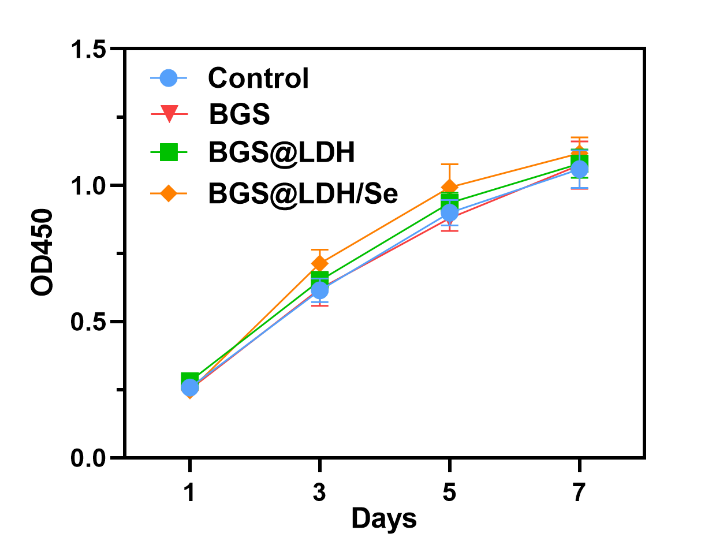


**Figure S17.** CCK-8 assays evaluating the biocompatibility of BGS, BGS@LDH, and BGS@LDH/Se for hBMSCs over 7 days.


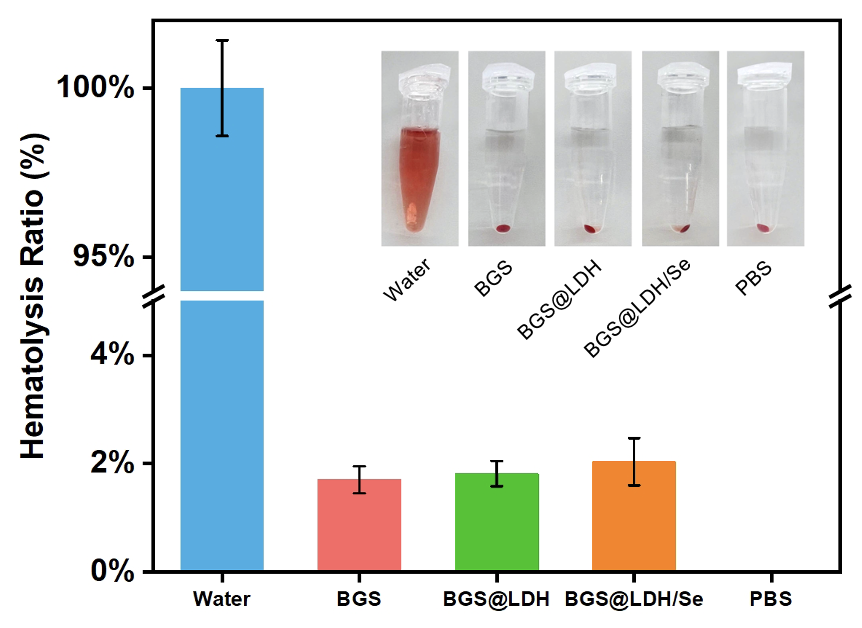


**Figure S18.** Hemolysis test was conducted using rabbit blood incubated with the leachates of BGS, BGS@LDH, or BGS@LDH/Se in comparison with positive control (incubated with water) and negative control (incubated with PBS).


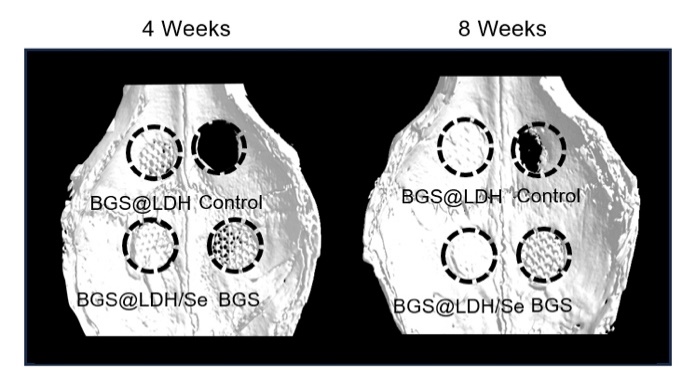


**Figure S19.** Posterior view of 3D reconstructed Micro-CT scanning of rabbit skull implanted with indicated scaffolds.

**
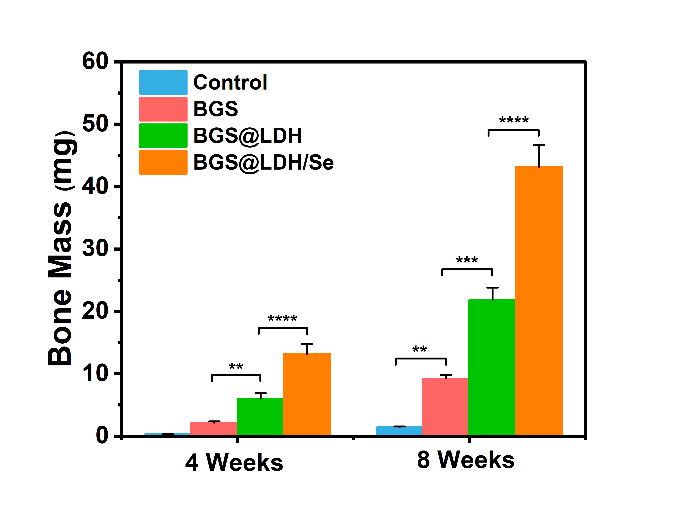
**

**Figure S20.** Quantitative analysis of the regenerated bone mass in control, BGS, BGS@LDH, and BGS@LDH/Se groups based on the 3D reconstructed Micro-CT images. Data are expressed as mean ± standard deviation (S.D.). Statistical comparisons were made by one-way ANOVA (for multiple comparisons): **p < 0.01, *** p < 0.001, **** p < 0.0001.


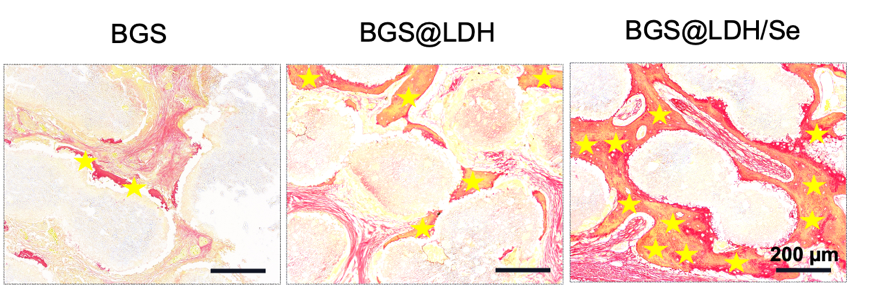


**Figure S21.** Sirius Red staining of new bone formation induced by BGS, BGS@LDH, and BGS@LDH/Se.

**
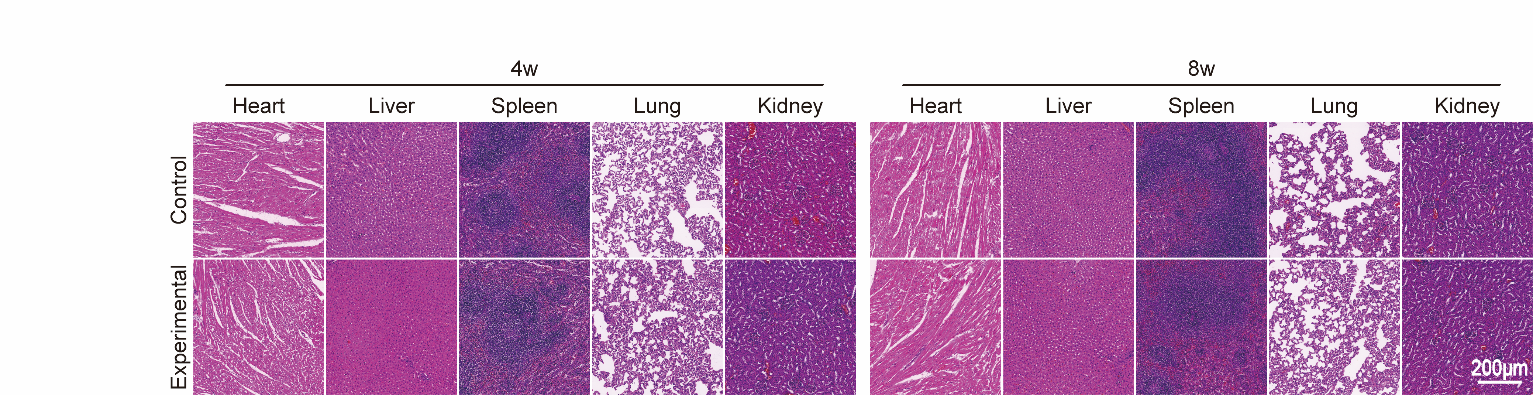
**

**Figure S22.** H&E staining of the heart, liver, spleen, lung, and kidney tissues of rabbits in control and experimental groups four and eight weeks after scaffold implantation.


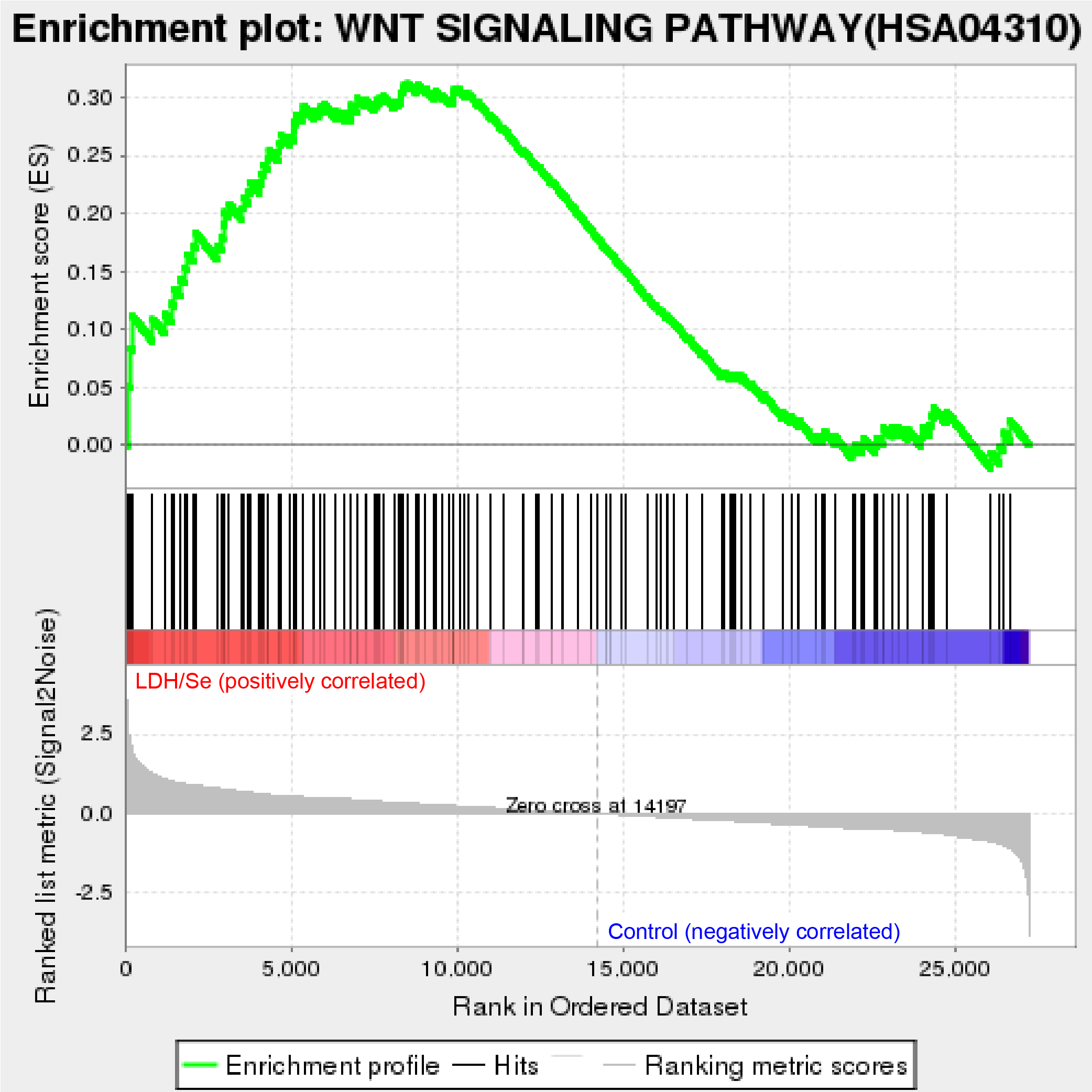


**Figure S23.** GSEA revealed that the gene set of the Wnt signaling pathway was positively correlated with BGS@LDH/Se intervention.

**
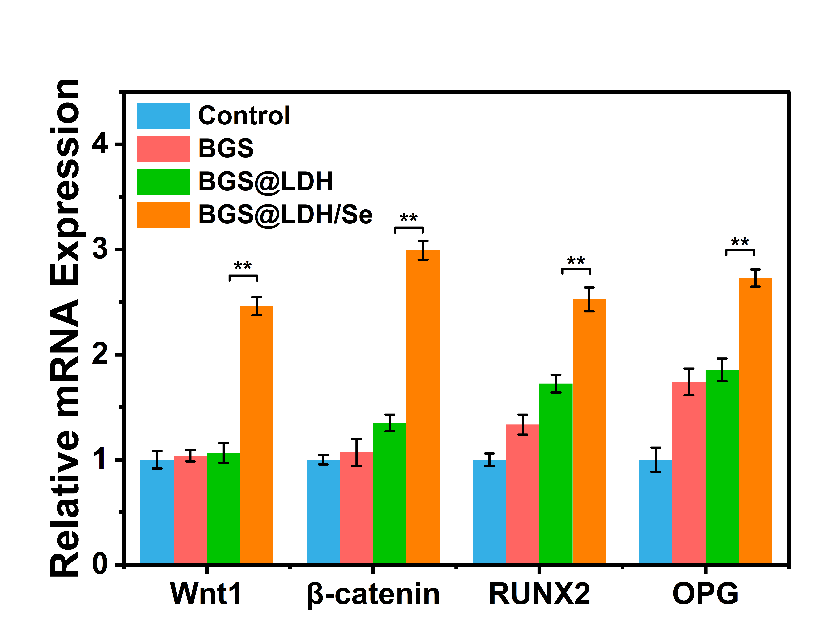
**

**Figure S24.** qPCR accessing the expression of genes involved in Wnt signal pathway (Wnt1, β-catenin, RUNX2, and OPG) of hBMSCs in control group or co-cultured with BGS, BGS@LDH, and BGS@LDH/Se.


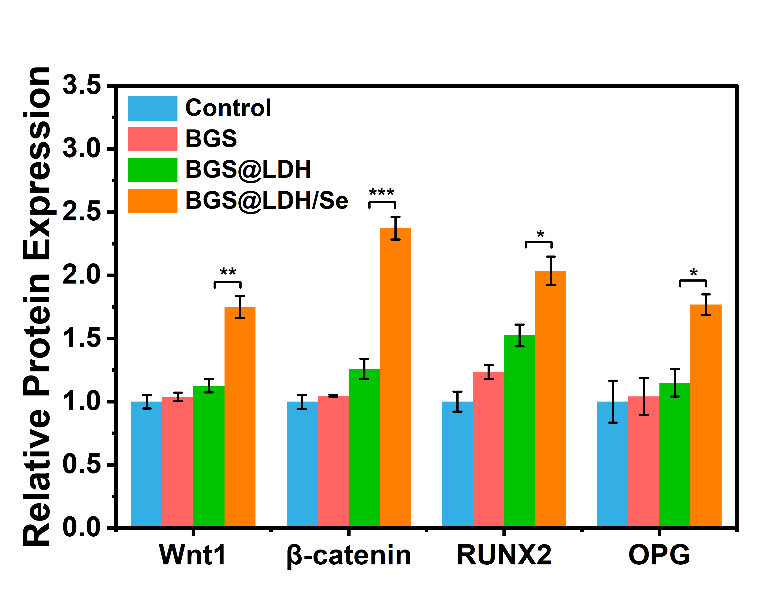


**Figure S25.** Quantitative analysis of the west blot bands in the control, BGS, BGS@LDH, and BGS@LDH/Se groups. Data are expressed as mean ± S.D. (n=3). Statistical comparisons were made by one-way ANOVA (for multiple comparisons): *p < 0.05, **p < 0.01, *** p < 0.001.

**
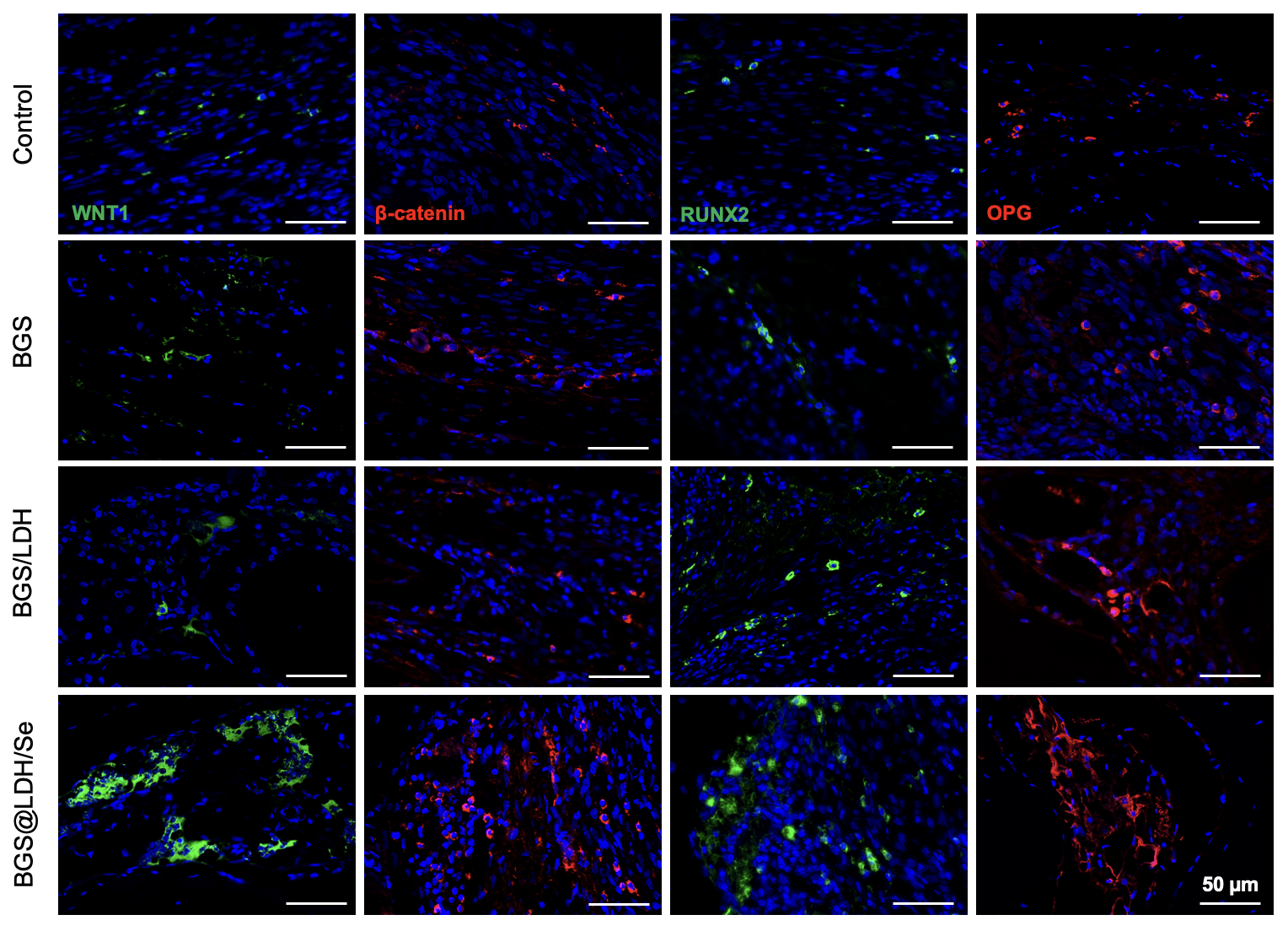
**

**Figure S26.** Immunofluorescence staining of Wnt1, β-catenin, RUNX2, and OPG in rabbit skull slides from the control, BGS, BGS@LDH, and BGS@LDH/Se areas.
